# Mobilizable plasmids drive the spread of antimicrobial resistance genes and virulence genes in *Klebsiella pneumoniae*

**DOI:** 10.1186/s13073-023-01260-w

**Published:** 2023-12-01

**Authors:** Jianfeng Zhang, Yanping Xu, Meng Wang, Xiaobin Li, Zhiyuan Liu, Dai Kuang, Zixin Deng, Hong-Yu Ou, Jieming Qu

**Affiliations:** 1 Department of Pulmonary and Critical Care Medicine, Ruijin Hospital, Shanghai Jiao Tong University School of Medicine, Shanghai, 200025 China; 2 State Key Laboratory of Microbial Metabolism, Joint International Laboratory on Metabolic & Developmental Sciences, School of Life Sciences & Biotechnology, Shanghai Jiao Tong University, Shanghai, 200030 China; 3 Institute of Antibiotics, Huashan Hospital, Fudan University, Shanghai, 200040 China; 4 Zhuhai Precision Medical Center, Zhuhai People’s Hospital (Zhuhai Hospital affiliated with Jinan University), Zhuhai, 519000 China; 5 National Health Commission (NHC) Key Laboratory of Tropical Disease Control, School of Tropical Medicine, Hainan Medical University, Haikou, China

**Keywords:** Mobilizable plasmid, Antimicrobial resistance gene, Virulence gene, CRISPR–Cas system, *Klebsiella pneumoniae*

## Abstract

**Background:**

*Klebsiella pneumoniae* is a notorious clinical pathogen and frequently carries various plasmids, which are the main carriers of antimicrobial resistance and virulence genes. In comparison to self-transmissible conjugative plasmids, mobilizable plasmids have received much less attention due to their defects in conjugative elements. However, the contribution of mobilizable plasmids to the horizontal transfer of antimicrobial resistance genes and virulence genes of *K. pneumoniae* remains unclear. In this study, the transfer, stability, and cargo genes of the mobilizable plasmids of *K. pneumoniae* were examined via genetic experiments and genomic analysis*.*

**Methods:**

Carbapenem-resistant (CR) plasmid pHSKP2 and multidrug-resistant (MDR) plasmid pHSKP3 of *K. pneumoniae* HS11286, virulence plasmid pRJF293 of *K. pneumoniae* RJF293 were employed in conjugation assays to assess the transfer ability of mobilizable plasmids. Mimic mobilizable plasmids and genetically modified plasmids were constructed to confirm the cotransfer models. The plasmid morphology was evaluated through *Xba*I and S1 nuclease pulsed-field gel electrophoresis and/or complete genome sequencing. Mobilizable plasmid stability in transconjugants was analyzed via serial passage culture. In addition, in silico genome analysis of 3923 plasmids of 1194 completely sequenced *K. pneumoniae* was performed to investigate the distribution of the conjugative elements, the cargo genes, and the targets of the CRISPR-Cas system. The mobilizable MDR plasmid and virulence plasmid of *K. pneumoniae* were investigated, which carry *oriT* but lack other conjugative elements.

**Results:**

Our results showed that mobilizable MDR and virulence plasmids carrying *oriT* but lacking the relaxase gene were able to cotransfer with a helper conjugative CR plasmid across various *Klebsiella* and *Escherichia coli* strains. The transfer and stability of mobilizable plasmids rather than conjugative plasmids were not interfered with by the CRISPR–Cas system of recipient strains. According to the in silico analysis, the mobilizable plasmids carry about twenty percent of acquired antimicrobial resistance genes and more than seventy-five percent of virulence genes in *K. pneumoniae.*

**Conclusions:**

Our work observed that a mobilizable MDR or virulence plasmid that carries *oriT* but lacks the relaxase genes transferred with the helper CR conjugative plasmid and mobilizable plasmids escaped from CRISPR–Cas defence and remained stable in recipients. These results highlight the threats of mobilizable plasmids as vital vehicles in the dissemination of antibiotic resistance and virulence genes in *K. pneumoniae*.

**Supplementary Information:**

The online version contains supplementary material available at 10.1186/s13073-023-01260-w.

## Background

The plasmid is a key carrier of crucial genes in bacterial pathogens such as those related to acquired antimicrobial resistance (AMR) and virulence. It plays an important role in horizontal gene transfer (HGT), greatly shaping bacterial ecology and evolution [1]. Plasmids are transferred in bacteria mainly through conjugation [2, 3]. Based on their transferable capacity and conjugation mechanism, plasmids can be categorized into three types: conjugative plasmids, mobilizable plasmids, and nonmobilizable plasmids [4, 5]. Conjugative plasmids are self-transmissible and contain the four necessary conjugative elements, including a complete type VI system (T4SS) gene cluster, the origin region of transfer (*oriT*), a type VI coupling protein (T4CP) gene, and a relaxase gene. Mobilizable plasmids that lack partial conjugative elements are nonself-transmissible and able to transfer with a helper conjugative plasmid [4, 6].

Conjugative and mobilizable plasmids account for approximately 50% of all plasmids [4, 7–9]. Conjugative plasmids are believed to be the most effective among plasmids in HGT due to their active and high-frequency transfer. Mobilizable plasmids are widely perceived as having small sizes with limited mobility and encoding at least a relaxase in addition to *oriT* [5, 7–10]. The transfer of mobilizable plasmids with only an *oriT* relies on the assistance of specialized conjugative plasmids that carry cognate relaxases capable of recognizing and cleaving *oriT* on mobilizable plasmids*.* The conditions required for achieving plasmid transfer are believed to be particularly demanding for these mobilizable plasmids [3, 9]. It has been noted that the plasmid transfer can be affected by bacterial defence systems against foreign DNA, such as the adaptive immune system CRISPR–Cas (clustered regularly interspersed short palindromic repeats-CRISPR-associated protein). The dissemination of conjugative plasmids in *Pseudomonas aeruginosa* and *Klebsiella pneumoniae* was restricted by the CRISPR–Cas system [11, 12]. The conjugative elements of these plasmids are often targeted by the CRISPR–Cas system [4, 11]. The contribution of mobilizable plasmids to the HGT of AMR and virulence genes might be significantly underestimated, particularly since mobilizable plasmids can be of large sizes and widely disseminated [7, 13, 14].

*K. pneumoniae* is a clinically alarming pathogen involved in multidrugresistance (MDR) and hypervirulence, serving as a major worldwide reservoir of AMR genes [15, 16]. Most acquired AMR genes and virulence genes in *K. pneumoniae* are plasmid-borne and transferred via conjugation [17–20]. In recent years, the conservative pLVPK-like virulence plasmid in hypervirulent *K. pneumoniae* (hvKP) has been increasingly identified in carbapenem-resistant *K. pneumoniae* (CRKP). The pLVPK-like virulence plasmid is a mobilizable plasmid, and its mobilization was proven to be the result of its interaction with the conjugative plasmid in our former work [11, 14]. However, the contribution of mobilizable plasmids to the HGT of AMR genes and virulence genes in *K. pneumoniae* is poorly understood.

Here, we examined the mobilizable plasmids in *K. pneumoniae* clinical isolates via genetic experiments and genomic analysis, focusing on their transfer, genetic stability, and cargo genes.

## Methods

### Bacterial strains and plasmids

CRKP HS11286, hvKP RJF293, *E. coli* C600, and other strains were employed to conduct conjugation assays. CR plasmid pHSKP2 and MDR plasmid pHSKP3 derived from CRKP HS11286, virulence plasmid pRJF293 derived from hvKP RJF293 were used to observe the plasmid transfer. Detailed information on the strains and plasmids used in this study is described in Additional file 1: Table S1. All strains used in this study were cultured at 37 °C in lysogeny broth (LB) medium (pH=7.2~7.4) with appropriate antibiotics.

### Gene editing and conjugation assays

We engineered genes in *K. pneumoniae* and *Escherichia coli* strains as previously described [21]. The primers used in this study are listed in Additional file 2: Table S2. Conjugative assays were performed as previously reported [14]. Overnight cultured donor strain and recipient strain were subcultured (1:100) in LB media at 220 rpm and 37 °C. Once the OD_600_ of the strains reached 0.6, 1 ml of donor cells and recipient cells were mixed (1:1), washed with PBS buffer, resuspended in 20 μl of 10 mM MgSO_4_, and incubated onto a 0.45 μm pore-size nitrocellulose filter placed on the surface of a prewarmed LB agar plate. After overnight culture at 37 °C, the strains were collected and resuspended in PBS buffer and diluted serially. The diluted bacteria were spread on LB agar plates containing the corresponding antibiotics. When *Klebsiella variicola* KvBSI002A was used as the recipient, 50 μg/ml apramycin was added to the plates. Plates containing 50 μg/ml rifamycin were employed when *E. coli* C600 was used as the recipient strain. To select transconjugants acquiring virulence plasmids, 10 μg/ml potassium tellurite (K_2_TeO_3_) was added to the plates. To track the transfer of the MDR plasmid, 50 μg/ml kanamycin was added to the plates. To track the transfer of the CR plasmid, 0.2 μg/ml meropenem was added to the plates. After overnight culture, colony-forming units (CFUs) on plates were enumerated. The transconjugants were validated and the conjugation frequency was calculated by dividing the number of correct transconjugants by the number of recipient cells. All conjugation experiments were conducted in two biological replicates with three parallel tests.

### Transconjugant verification

The antibiotic resistance of transconjugants obtained in the conjugation assays was primarily validated via replica plating bacteria through stretching single colonies on LB agar plates and those supplied with appropriate antibiotics. A single clone of these bacteria on LB agar plates with antibiotics was picked and verified via PCR with specific primers. The correct transconjugants were further validated by *Xba*I and S1 nuclease pulsed-field gel electrophoresis (*Xba*I and S1 PFGE) to study the morphology of plasmids in transconjugants.

### Detection of plasmid stability in the transconjugant

A single colony of each purified strain was picked from the freshly streaked LB agar plate and inoculated into fresh LB broth. After being cultured overnight at 37 °C, 4 μl of the bacterial suspension was reincubated in 4 ml of fresh LB broth. The process was repeated, and the strain was reincubated in serial culture. The plasmid stability was assessed by two steps: (i) streaking each subculture of the transconjugants on fresh LB agar plates; and (ii) randomly selecting three single colonies for antibiotic resistance verification and PCR detection of the specific gene on the corresponding plasmids. The number of clones carrying the plasmids was counted and recorded. If none of the three clones harbor a plasmid, it was recorded as zero, indicating plasmid loss. S1-PFGE was performed for transconjugants with fusion plasmids to visualize the morphological characteristics of plasmids as previously reported [14].

### Complete genome sequencing and annotation

The genomic DNA of transconjugants *E. coli* C600-p2-V and C600-p2-3 was extracted and sequenced by using the combination of the 150-bp paired-end Illumina NovaSeq 6000 platform and the PacBio RSII single-molecule long-read sequencing platform. Then the genome sequence was assembled, annotated, and deposited with the GenBank databases as previously reported [14]. Canu 2.0 [22] was used for the de novo assembly of the trimmed and filtered reads. The genome sequences of transconjugants are deposited in the National Center for Biotechnology Information (NCBI) BioProject repository, with the accession number PRJNA903763 (https://www.ncbi.nlm.nih.gov/bioproject/PRJNA903763/) [23]. The genomic data were annotated with Prokka 1.1.3 [24]. The ISfinder web server [25] was used to detect the insertion sequence (IS) with default parameters in fusion plasmids. Plasmid sequences were aligned and visualized using the BLAST Ring Image Generator (v0.95) [26] and Easyfig (v2.2.2) [27].

### Cargo gene analysis of GenBank-archived plasmid genomes

A total of 1194 completely sequenced *K. pneumoniae* strains containing 3,923 plasmids were downloaded from GenBank by September 22, 2021 (Additional file 3: Table S3). We utilized VRprofile2 [28] and Kleborate (v2.0.4) [29] with default parameters to detect the multilocus sequence typing (MLST), AMR genes and virulence genes, and oriTfinder (v1.0) with default parameters [30] to detect conjugation elements comprising T4SS, T4CP, *oriT*, and relaxase. The AMR genes include genes that confer resistance to aminoglycoside, colistin, fosfomycin, macrolide, phenicol, rifampin, sulfonamide, tetracycline, trimethoprim, and beta-lactam. The plasmids containing at least three categories of AMR genes are denoted as the MDR plasmid [17, 31]. The plasmid containing the carbapenem resistance gene is defined as the CR plasmid. The virulence genes include the *iuc* cluster, *rmpADC* cluster, and *rmpA2*, which could confer hypervirulence to *K. pneumoniae* [16, 32–34]. To quantify the virulence genes, the full length of the *iuc* cluster or *rmpADC* cluster was summed as the units of virulence genes. The plasmids containing the *rmpA2*, or *rmpADC* gene cluster, and the *iuc* gene cluster were defined as virulence plasmids as previously reported [14, 35, 36]. The plasmid incompatibility groups of 3923 plasmids were divided into 19 types from IncFIB to Co1156 via PlasmidFinder (v2.0.1) [37] with default parameters. The replicons that could not be identified were classified as “Others”. The coexistence of plasmids was visualized by networkx (v3.1) [38] and matplotlib [39]. iTOL (v6.1) [40] was used to map the distribution of genes in plasmids.

### Classification of plasmids according to mobility ability

A T4SS containing at least 12 nonrepeated T4SS structural proteins is defined as intact and functional. The relaxases detected by oriTfinder [30] were classified based on MOB families using HMMER v.3.1 against the conjugation database of MacSyFinder 2.0 [41] applying an *E*-value < 1E^−5^. The alignment of short core *nic* sequences was conducted by pairwise comparison of all *oriT*s detected in *K. pneumoniae* with manual curation. According to the conjugative mechanism [3, 4], plasmids containing a complete set of conjugative elements, including functional T4SS, T4CP, relaxase, and *oriT* are defined as conjugative plasmids. Plasmids that possess an *oriT* but lack an intact T4SS gene cluster, T4CP, or relaxase are classified as mobilizable plasmids. Except for conjugative plasmids and mobilizable plasmids, the remaining plasmids are regarded as nonmobilizable.

### Detection of the CRISPR–Cas system and proto-spacers on plasmids

CRISPRloci (v1.0.0) [42] was used to identify the CRISPR-Cas systems (including the CRISPR loci and spacers) with default parameters in the *K. pneumoniae* genome sequences. The anti-CRISPR proteins were detected using a previously reported model [43]. The detected spacer sequences of CRISPR-Cas systems were used to search proto-spacers, the targets of CRISPR–Cas systems, on the plasmid sequences via BLASTn with a minimum of 90% identities, and only CRISPR arrays with 3 spacers or more were used in our analysis. The spacers were clustered according to 90% nucleotide sequence identities and proto-spacers were grouped based on their nearest genes. Information on CRISPR–Cas systems and their proto-spacers is listed in Additional file 4: Table S4.

### Bacterial growth curve and competition assays

For the bacterial growth curve analysis, overnight cultures of recipient strains and their transconjugants were inoculated into LB medium at a dilution of 1:1000. The cultures were then incubated at 37 °C and 220 rpm. The OD_600_ was measured with three repeats every half hour using a spectrophotometer to monitor the growth of the bacteria over time.

In the competition assays, after overnight culturing, 1 ml of equal amounts of the different strains (recipient and transconjugant) were mixed together. The mixed culture was then washed with PBS buffer to remove any residual media. The bacterial cells were subsequently resuspended in 20 μl of PBS and spread onto the surface of prewarmed LB agar. After 24 h of competition, the bacterial plaques were resuspended in 1 ml of PBS and then subjected to gradient dilution. The appropriate dilutions were plated onto LB agar supplemented with the corresponding antibiotics. The plates were incubated overnight, and CFUs of each strain were enumerated.

## Results

### Natural mobilizable MDR and virulence plasmids lacking the relaxase gene transferred with the helper conjugative CR plasmid

**Fig. 1:**
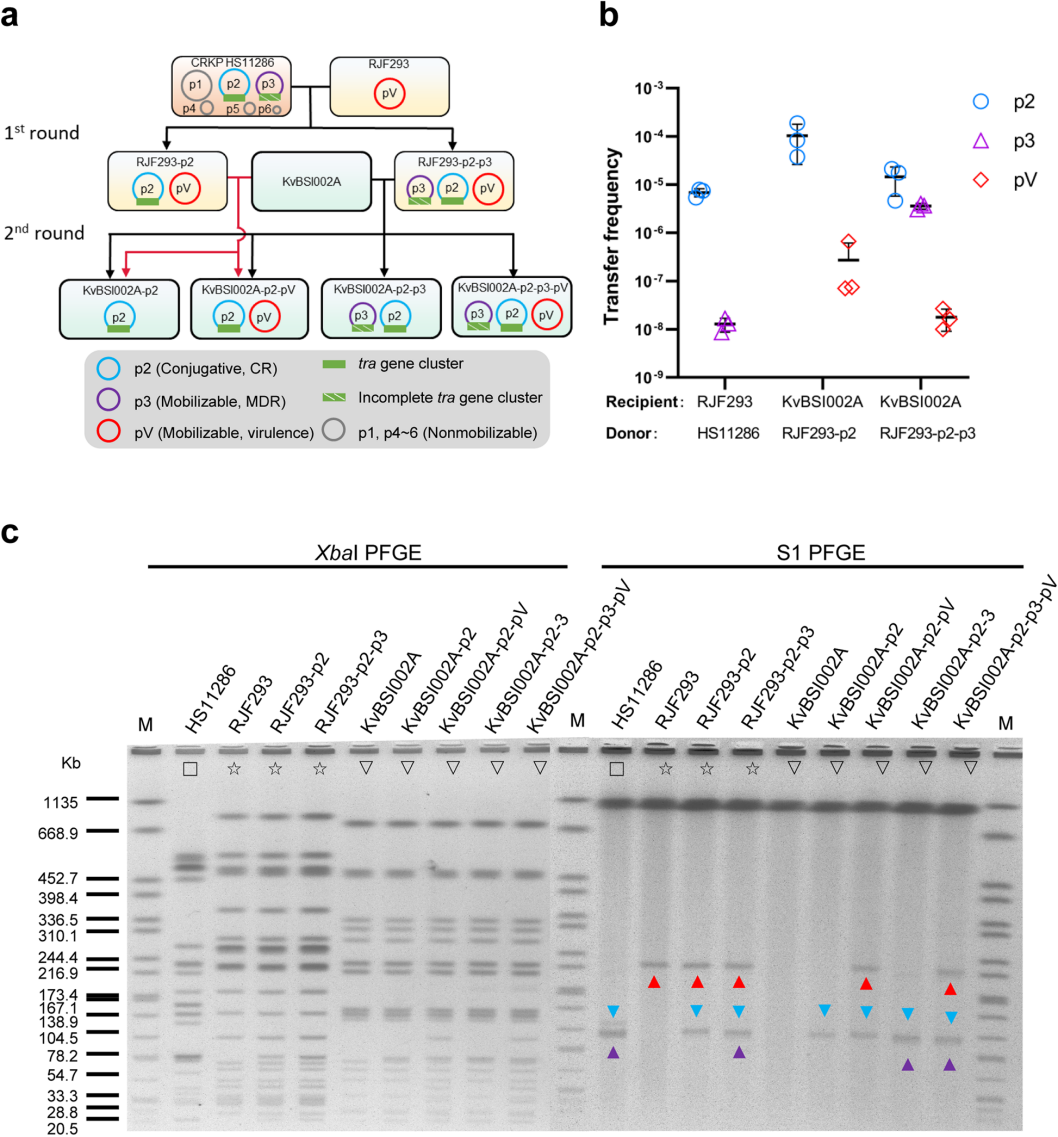
Transfer of the mobilizable MDR plasmid pKPHS3 (p3) and the virulence plasmid pRJF293 (pV), both lacking relaxase genes, with the help of the conjugative CR plasmid pKPHS2 (p2). **a** Schematic diagram of the two rounds of conjugation assays. The green square denotes the *tra* gene cluster on pKPHS2. The green square with white slashes denotes the incomplete *tra* gene cluster on pKPHS3. The red line indicates the transconjugants generated from *K. pneumoniae* RJF293-p2 conjugated with *K. variicola* KvBSI002A. **b** The conjugation frequencies of pKPHS2, pKPHS3 and pRJF293. The donor strains were HS11286, RJF293-p2, and RJF293-p2-p3. The recipient strains were RJF293 and KvBSI002A. Detailed data are available in Additional file 6: Table S5. **c** *Xba*I PFGE and S1-PFGE of transconjugants and their parental strains. M represents the molecular weight marker *Salmonella* serotype *Braenderup* H9812. Strains with the same symbol on the PFGE image represent the progeny derived from the same parental strain. The red lines present the transconjugants generated from RJF293-p2 with KvBSI002A. The CR plasmid, MDR plasmid, and virulence plasmid on the S1-PFGE image are indicated by blue triangles, purple triangles, and red triangles, respectively. The plasmids and strains were further confirmed by PCR

**Fig. 2:**
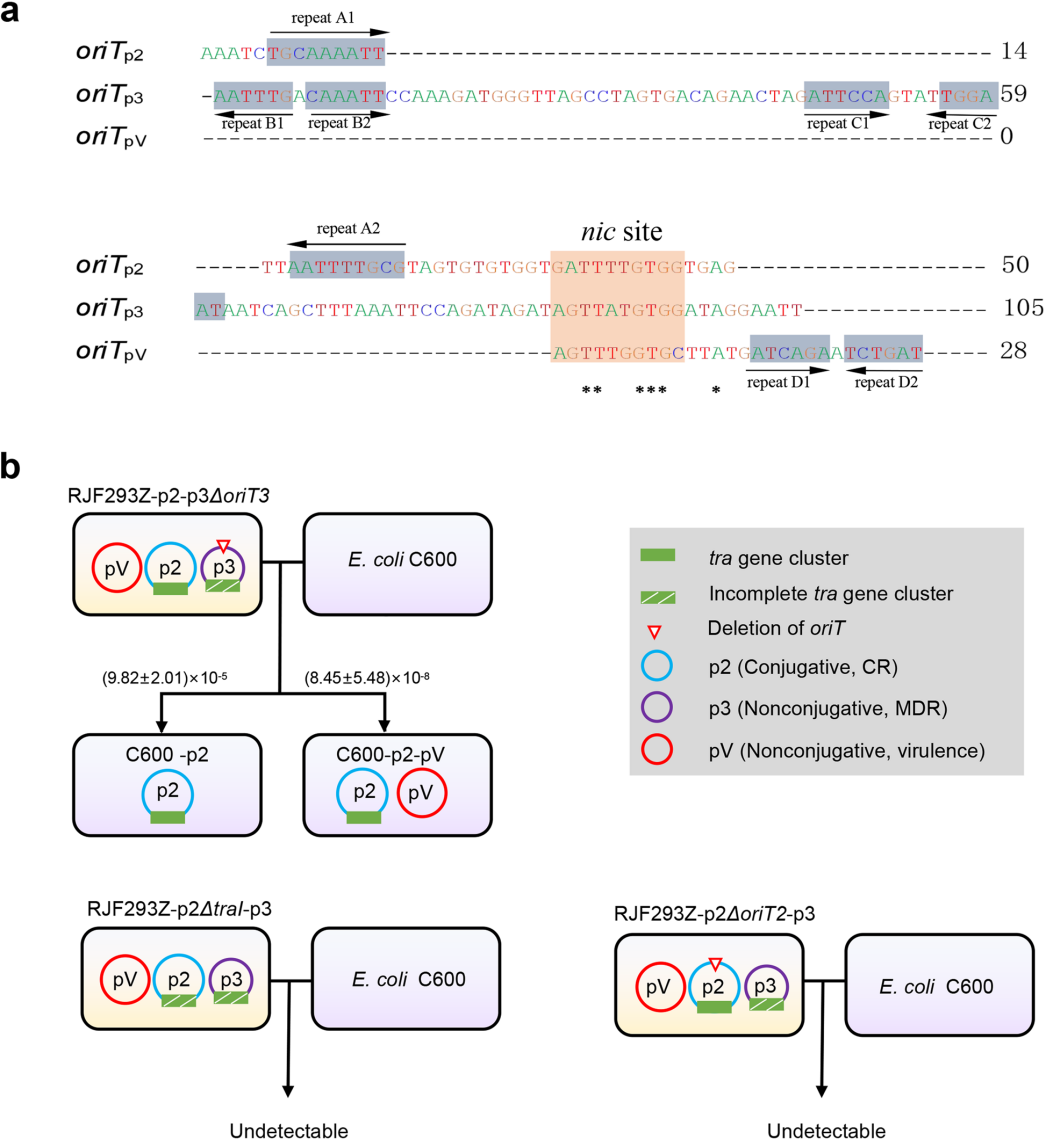
Conjugation assays of gene-edited plasmids. **a** Alignment of the *oriT*s of pKPHS2 (p2), pKPHS3 (p3), and pRJF293 (pV). **b** Schematic diagram of conjugation assays for pKPHS2, pKPHS3, pRJF293 and their nonconjugative derivatives. The green square denotes the *tra* gene cluster on pKPHS2 (p2). The green square with white slashes denotes the incomplete *tra* gene cluster on pKPHS3 (pV). The red inverted triangle presents the deletion of the *oriT* on plasmids. The conjugative frequencies of detected transconjugants that acquired either pKPHS2 (p2) alone or both pKPHS2 (p2) and pRJF293 (p3) are listed. Detailed data are available in Additional file 6: Table S5

The clinical CRKP isolate HS11286 [44] contains 6 plasmids, including pKPHS1~6 (Fig. 1). The CR IncFII plasmid pKPHS2 (p2) is supposed to be a self-transferable conjugative plasmid, as it carries an *oriT*, an intact *tra* gene cluster, a MOB_F_ relaxase gene, and a T4CP gene. The MDR IncA/C plasmid pKPHS3 (p3) is expected to be a nonself-transmissible but mobilizable plasmid, as it lacks the relaxase gene and a complete *tra* gene cluster but contains an *oriT*. The other four plasmids, pKPHS1 and pKPHS4~6, are regarded as nonmobilizable. Interestingly, pKPHS3 contains an *oriT* that shares a similar core *nic* site with the conjugative plasmid pKPHS2, similar to the mobilizable virulence plasmid pRJF293 (pV) [14] in the K2 hvKP RJF293 (Fig. 2 a). To experimentally investigate the transfer of the conjugative CR plasmid pKPHS2, the mobilizable MDR plasmid pKPHS3 and the mobilizable virulence plasmid pRJF293, we employed CRKP HS11286 to conjugate with hvKP RJF293 and the plasmid-free *Klebsiella variicola* strain KvBSI002A. In the first round of the conjugation assay, pKPHS2 was transferred from the donor HS11286 to the recipient RJF293 at a frequency of (6.89±1.34)×10^−6^, and pKPHS3 was transferred at a frequency of (1.29±0.41)×10^−8^ (Fig. 1; Additional file 6: Table S5). In the second round, pKPHS2 was further transferred into the recipient *K. variicola* KvBSI002A alone or cotransferred with pKPHS3 and/or pRJF293 (Fig. 1; Additional file 6: Table S5). In this conjugation assay, the transfer of nonmobilizable pKPHS1 and pKPHS4~6 was not detected as expected.

To observe the dynamic cotransfer of the mobilizable MDR plasmid pKPHS3 with the conjugative CR plasmid pKPHS2, we selected three *K. pneumoniae* strains to conduct conjugative assays. The results showed that pKPHS3 can transfer with pKPHS2 from ST11 CRKP HS11286 into K1 hvKP RJF999 and further transfer into K2 hvKP RJF293H (Additional file 5: Fig. S1; Additional file 6: Table S5). The same phenomenon was observed in the mobilizable virulence plasmid pRJF293 (Additional file 5: Fig. S2; Additional file 6: Table S5). When K2 hvKP RJF293-p2 or K1 hvKP RJF999-p2 served as the donor to conjugate with *K. variicola* KvBSI002A, the mobilizable virulence plasmids were transferred into *K. variicola* KvBSI002A with the accompaniment of pKPHS2, and further transferred into CRKP HS11286. The cotransfer of mobilizable MDR or virulence plasmids with CR plasmids across different strains generated a series of transconjugants, such as hypervirulent carbapenem-resistant *K. pneumoniae* (hv-CRKP), and hypervirulent multidrug-resistant carbapenem-resistant *K. pneumoniae* (hv-MDR-CRKP).

We also examined the cotransmission of the mobilizable MDR plasmid pKPHS3 and virulence plasmid pRJF293 with the conjugative CR plasmid pKPHS2 from *K. pneumoniae* to *E. coli*. A typical capsule-free strain, *E. coli* C600 [45], was employed as the recipient. We found that both mobilizable plasmids pRJF293 and pKPHS3 can transfer with conjugative pKPHS2 from *K. pneumoniae* RJF293-p2-p3 into *E. coli* C600. Among *E. coli* transconjugants, the two mobilizable plasmids stayed separate, or formed hybrid plasmids with pKPHS2 (Additional file 5: Fig. S3; Additional file 6: Table S5). The PFGE and WGS results showed that in transconjugants, pKPHS2 and pKPHS3 formed a 196,720 bp hybrid plasmid p2-3 at the edge of a Tn*3* IS family. The fusion event might occur via replicative transposition or homologous recombination (Additional file 5: Fig. S4). In transconjugant C600-p2-V, there were two hybrid plasmids, including a 264,857 bp plasmid p2-V and a 74,014 bp plasmid p2-V-2. These hybrid plasmids speculatively underwent two rounds of recombination events to form a large fusion plasmid derived from pKPHS2 and pRJF293, followed by division into two separate plasmids (Additional file 5: Fig. S5). Since the timing of plasmid fusion events cannot be detected by PFGE or WGS during conjugation, it is unclear whether the fusion occurred before or after plasmid transfer. However, these fusion plasmids remained stable in passage cultures, as confirmed by S1-PFGE (Additional file 5: Fig. S6). We speculate that plasmid cointegration occurred after plasmid transfer and was aimed at adapting the *E. coli* host, which needs to be verified in the future.

### The transfer of mobilizable plasmids depended on their *oriT* and the aid of the helper conjugative plasmid

To investigate the transmission of the mobilizable MDR pKPHS3 (p3) and the virulence pRJF293 (pV) with their *oriT*s (Fig. 2 a) and the conjugative CR pKPHS2 (p2), we deleted the relaxase-encoding gene *traI* from pKPHS2, and then pKPHS2*ΔtraI* lost its transferable ability. The transfer of pKPHS3 and pRJF293 was also not detected. We also deleted the *oriT* from pKPHS3, and pKPHS3*ΔoriT3* also lost its mobilizable ability (Fig. 2 b; Additional file 6: Table S5). This indicated that the mobility of the MDR plasmid pKPHS3 relied on its *oriT* and the helper CR plasmid pKPHS2*.*

**Fig. 3:**
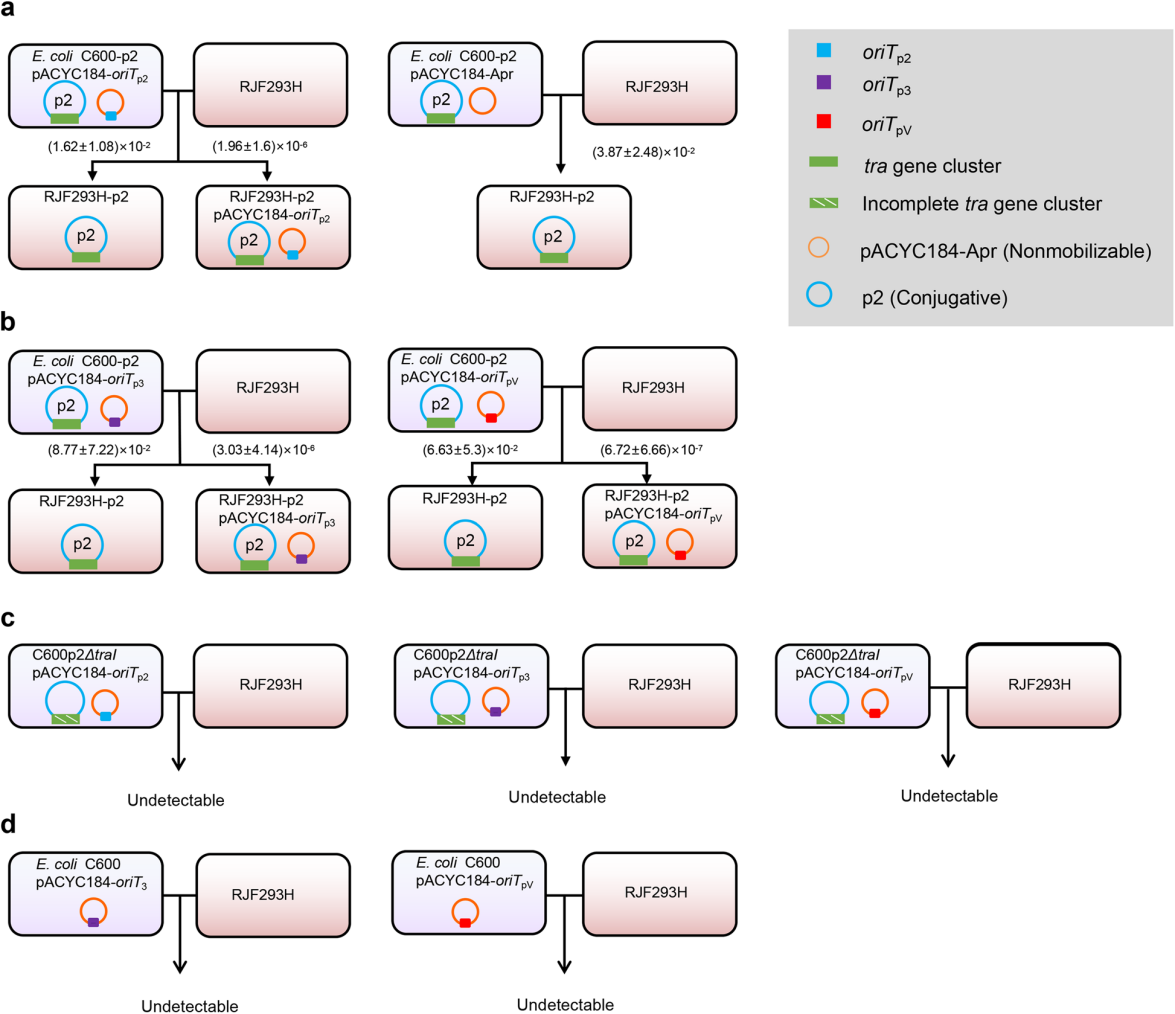
Schematic diagram of the simulation of mobilization of the virulence plasmid pRJF293 (pV) and the MDR plasmid pKPHS3 (p3) with their *oriT*s and the help of the CR plasmid pKPHS2 (p2). **a** When accompanied by pKPHS2, pACYC184-*oriT*_p2_ can be transferred while the empty vector pACYC184 cannot. **b** When accompanied by plasmid pKPHS2, pACYC184-*oriT*_p3_ and pACYC184-*oriT*_pV_ can be transferred. **c** pACYC184-*oriT*_p2_, pACYC184-*oriT*_p3_, and pACYC184-*oriT*_pV_ cannot be transferred when accompanied by plasmid pKPHS2*ΔtraI*. **d** pACYC184-*oriT*_p3_ and pACYC184-*oriT*_pV_ cannot be transferred alone. The orange circle denotes the backbone of plasmid pACYC184-Apr, and the blue circle denotes the conjugative plasmid pKPHS2. The green square denotes the *tra* gene cluster. The blue square denotes the *oriT* derived from plasmid pKPHS2. The purple square denotes the *oriT* derived from pKPHS3. The red square denotes the *oriT* derived from pRJF293. The conjugative frequencies of detected transconjugants that acquired either pKPHS2 (p2) or both pKPHS2 (p2) and pACYC184 derivatives are listed. Detailed data are available in Additional file 6: Table S5

We then cloned *oriT*s of pKPHS2, pKPHS3, and pRJF293 into the vector pACYC184-Apr, respectively. These constructed *oriT-*containing plasmids rather than the empty vector pACYC184-Apr were transferred from *E. coli* C600 into *K. pneumoniae* RJF293H when they coexisted with conjugative pKPHS2. Meanwhile, they failed to transfer when the helper plasmid pKPHS2 was absent. These plasmids were also not transferred under the accompaniment of pKPHS2*ΔtraI* (Fig. 3; Additional file 6: Table S5). The results showed that the transfer of mobilizable plasmids depended on their *oriT*s and the aid of the helper conjugative plasmid pKPHS2. Several reports proposed that the transfer of mobilizable plasmids lacking the relaxase gene might require the other plasmids to provide relaxases [3, 46]. Here, we experimentally validated that natural mobilizable MDR or virulence plasmids lacking the relaxase gene in the *K. pneumoniae* clinical isolates could still be transferred with the aid of the helper conjugative CR plasmid.

### Mobilizable plasmids were not affected by the CRISPR–Cas system in recipients

**Fig. 4:**
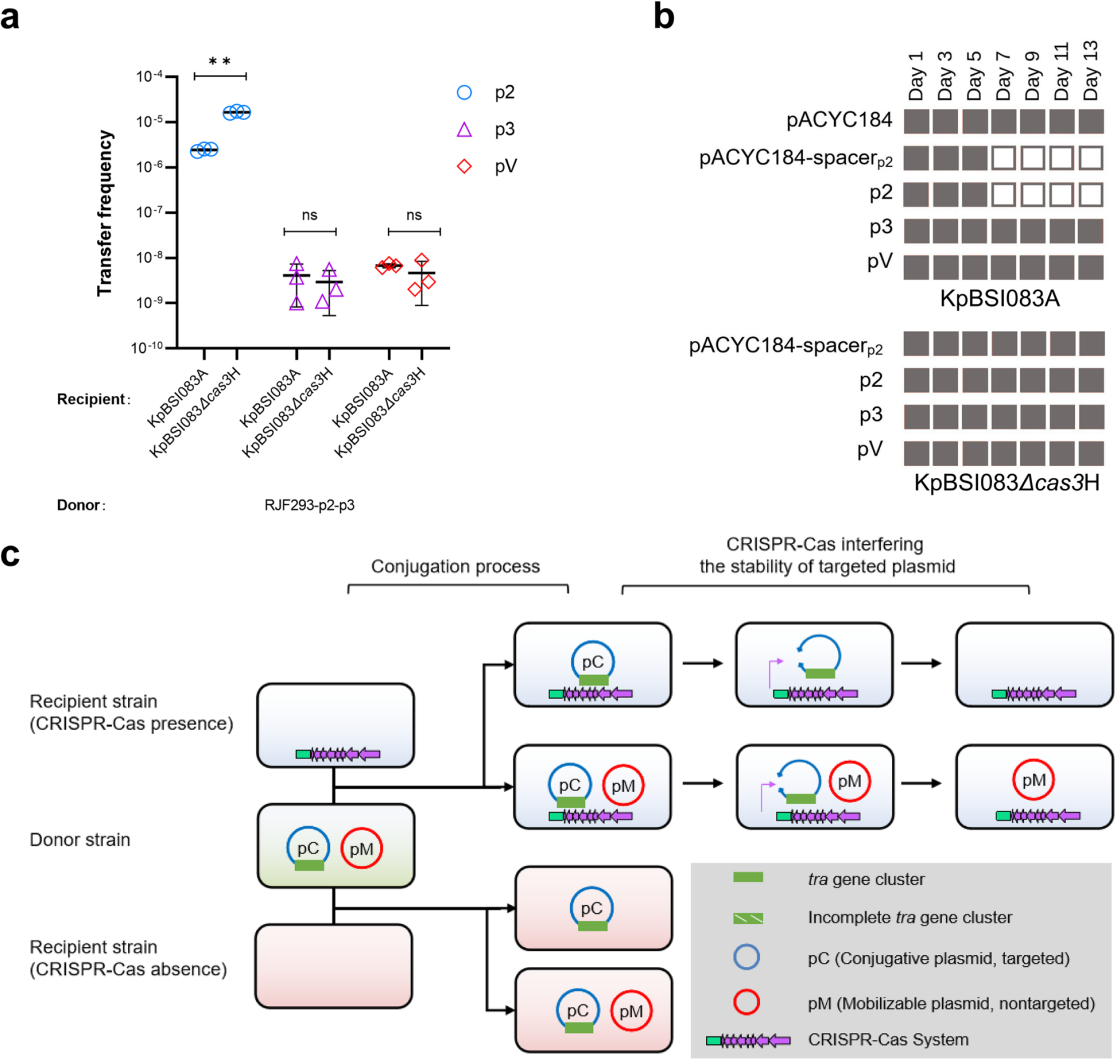
Effect of the CRISPR–Cas system in recipient strains on the stability of mobilizable plasmids. **a** The conjugation frequencies of the targeted conjugative pKPHS2 (p2), the nontargeted mobilizable pKPHS3 (p3) and pRJF293 (pV). The donor strain was *K. pneumoniae* RJF293-p2-p3 and the recipient strains were *K. pneumoniae* KpBSI083A and KpBSI083*Δcas3*H. Detailed data are available in Additional file 6: Table S5. **b** Schematic of plasmid stability in transconjugants during 13 days of serial passage culture. The plasmid was detected by PCR and antimicrobial susceptibility tests. Plasmids that were retained are indicated by gray squares, while plasmids that were lost are indicated by white squares. Full details of plasmid stability in transconjugants are shown in Additional file 7: Table S6. **c** The proposed mode of the impact of the CRISPR–Cas system on the dissemination of targeted conjugative plasmids

We found that the conjugative CR plasmid pKPHS2 carries a proto-spacer targeted by the CRISPR–Cas system of the ST15 plasmid-free *K. pneumoniae* strain KpBSI083A. However, the mobilizable plasmids pKPHS3 and pRJF293 both do not. To study the impact of the CRISPR–Cas system on the transfer of its targeted conjugative plasmid and nontargeted mobilizable plasmids, we used transconjugants RJF293-p2 and RJF293-p2-p3 as donors to conjugate with *K. pneumoniae* KpBSI083A and its derivative KpBSI083*Δcas3*H, which lacks the core nuclease Cas3 of the CRISPR–Cas system. The conjugation assays showed that the transfer of pKPHS2 into KpBSI083A occurred at a significantly higher frequency than its transfer into KpBSI083*Δcas3*H [(1.66±0.09)×10^-5^ versus (2.44±0.17)×10^-6^, *p* = 0.0000097]. Both mobilizable plasmids pKPHS3 and pRJF293 were transferred into KpBSI083A at frequencies comparable with their transfer into KpBSI083*Δcas3*H [(4.11±3.29)×10^−9^ versus (2.92±2.38)×10^-9^, *p* = 0.90 and (6.78±0.69)×10^−9^ versus (7.67±3.77)×10^−9^, *p *= 0.21], respectively (Fig. 4 a; Additional file 5: Fig. S7; Additional file 6: Table S5). The results showed that the CRISPR–Cas system in the recipient strain could restrain the acquisition of its targeted conjugative plasmid pKPHS2 but not the nontargeted mobilizable plasmids pKPHS3 and pRJF293.

We then examined the genetic stability of these plasmids in the transconjugants of *K. pneumoniae* KpBSI083A and KpBSI083*Δcas3*H. We constructed a vector, pACYC184-spacer_p2_, containing the same proto-spacer as pKPHS2, which was targeted by the CRISPR–Cas system of *K. pneumoniae* KpBSI083A. After approximately 7 days of serial passage culture without the addition of additional antibiotics, pKPHS2, and the vector pACYC184-spacer_p2_ were lost in the host strain KpBSI083A. The loss of the plasmid was confirmed by PCR and antibiotic susceptibility tests. Nontargeted pKPHS3, pRJF293, and empty vector pACYC184-Apr all remained stable in KpBSI083A after 13 days of serial passage culture (Fig. 4 b, Additional file 7: Table S6). Meanwhile, regardless of whether the plasmid contained the protospacer, the plasmids all remained stable in KpBSI083*Δcas3*H in 13-day serial culture (Additional file 7: Table S6). The results showed that the CRISPR–Cas system in the recipient could interfere with the genetic stability of its targeted conjugative plasmid, while the mobilizable plasmids without proto-spacer could be free from interference (Fig. 4 c).

The plasmid distribution in transconjugants also corresponds with the conjugation assay results. When KpBSI083A served as the recipient, we detected two types of new transconjugants, KpBSI083A-p3 and KpBSI083A-pV, which only contained either the mobilizable MDR plasmid pKPHS3 or the virulence plasmid pRJF293. The corresponding transconjugants with only the mobilizable plasmid were not observed when KpBSI083*Δcas3*H served as the recipient (Additional file 5: Fig. S7). The transconjugants KpBSI083A-p3 and KpBSI083A-pV might result from the degradation of pKPHS2 in KpBSI083A-p2-p3 and KpBSI083A-p2-pV, which is caused by the CRISPR–Cas system (Additional file 5: Fig. S8).

We further compared the proto-spacers of the CRISPR–Cas system in mobilizable plasmids and conjugative plasmids of *K. pneumoniae* to explore the effect of CRISPR–Cas restriction on the transfer of mobilizable plasmids. We compiled a dataset of 1194 completely sequenced *K. pneumoniae* genomes containing 3923 plasmids from GenBank (Additional file 6: Table. S5). According to the conjugative mechanism and plasmid mobility, 3,923 plasmids were divided into conjugative plasmids (*n *= 1073), mobilizable plasmids (*n *= 811), and nonmobilizable plasmids (*n *= 2039). In 1194 *K. pneumoniae* strains, 297 CRISPR–Cas systems were identified and mainly distributed in the sequence types (STs) ST15, ST23, ST147, ST14, and ST45 (Additional file 5: Fig. S9). Most of the CRISPR–Cas systems belong to Types I-E (*n *= 198), while a few are classified as Types IV (*n *= 48), V (*n *= 46), and VI (*n *= 4), consistent with previous reports [47–50]. According to sequence similarity, spacers can be divided into 917 unique spacers, including 184 spacers targeting plasmids in *K. pneumoniae*. Of the proto-spacers located on plasmids, 62.8% (12,922/20,580) and 28.2% (5798/20,580) were found on conjugative plasmids and nonmobilizable plasmids, respectively, while only 9.0% (1860/20,580) were carried by mobilizable plasmids (Additional file 4: Table S4; Additional file 5: Fig. S10). This indicated that the mobilizable plasmids with fewer proto-spacers might have a larger potential capacity to disseminate with a wider host range than conjugative plasmids.

### Mobilizable plasmids and conjugative plasmids in *K. pneumoniae* enriched AMR and virulence genes

**Fig. 5:**
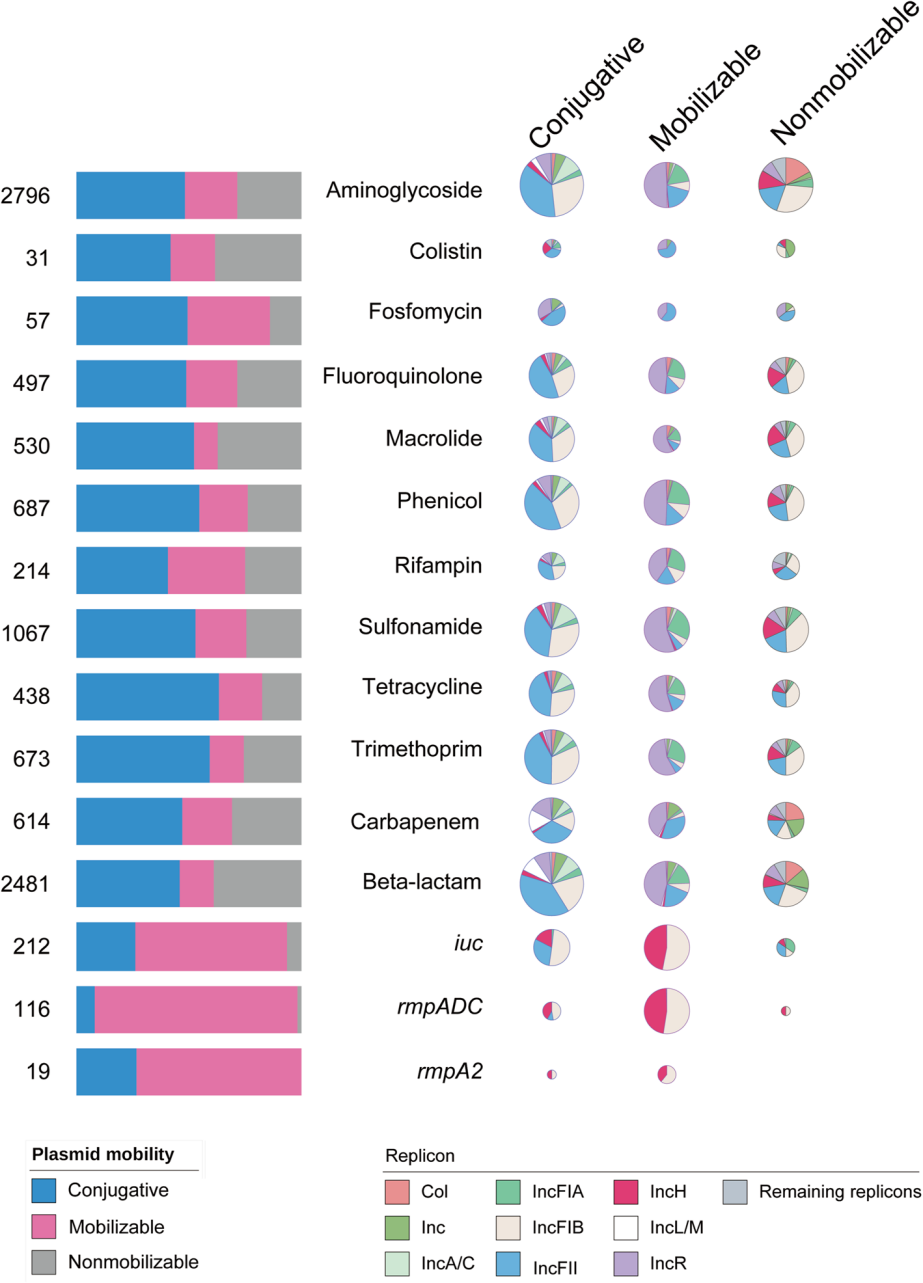
Distribution of AMR genes and virulence genes in *K. pneumoniae* on plasmids with different transmissible abilities. The 1194 completely sequenced *K. pneumoniae* genomes containing 3923 plasmids were taken from GenBank. The left bars represent the number of AMR genes or virulence genes and their distribution on plasmids with different mobilities. The replicon distribution in plasmids carrying different AMR genes or virulence genes with different mobilities is illustrated via pie charts. Inc. in Replicon presents the sum of replicons ranging from IncI to IncX. Col. in Replicon represents the sum of replicons ranging from ColRNAI to Col156. The remaining replicons represent the unknown replicons detected by PlasmidFinder

A number of mobilizable plasmids and conjugative plasmids, including those of considerable size, were distributed in *K. pneumoniae* of six typical STs from ST11 to ST147 (Additional file 5: Fig. S11). After in silico analysis of the cargo protein-coding genes in 3923 plasmids of 1194 completely sequenced *K. pneumoniae* genomes, 87.1% (9545/10,954) of AMR genes and 86.8% (347/400) of virulence genes were located on plasmids (Additional file 3: Table S3). In these plasmid-borne genes, 50.1% (4781/9545), 21.4% (2040/9545), and 28.5% (2724/9545) of AMR genes were clustered on conjugative plasmids, mobilizable plasmids, and nonmobilizable plasmids, respectively. In addition, 75.8% (263/347) and 19.9% (69/347) of virulence genes were carried by mobilizable plasmids and conjugative plasmids, respectively (Fig. 5; Additional file 3: Table S3; Additional file 5: Fig. S9). Only 4.3% (15/347) of virulence genes were located on nonmobilizable plasmids. The ST11 clonal strains [17] were the most predominant *K. pneumoniae* isolates in China and contained these resistance and/or virulence plasmids (Additional file 5: Fig. S12). In ST11 strains, 30.1% (217/720) of plasmids were conjugative and 32.9% (237/720) were mobilizable (Additional file 3: Table S3; Additional file 5: Fig. S9). The above results suggested that in *K. pneumoniae*, conjugative plasmids, mobilizable plasmids, and nonmobilizable plasmids all carry AMR genes. Meanwhile, mobilizable plasmids are the main carriers of acquired virulence genes in *K. pneumoniae*.

As the transfer of mobilizable plasmids requires the aid of conjugative plasmids, we also examined the coexistence of the mobilizable and conjugative plasmids in 1194 completely sequenced *K. pneumoniae* strains. Of the mobilizable plasmids, 68.7% (557/811) were accompanied by conjugative plasmids (Additional file 5: Fig. S13a). In *K. pneumoniae* strains belonging to STs associated with the top ten highest number of plasmids, approximately 72.4% of mobilizable plasmids coexisted with conjugative plasmids (Additional file 5: Fig. S13b). The plasmid pattern of prevailing and clinically worrying CRKP strains was further surveyed. Among 537 CRKP strains, 70.5% (423/600) of CR plasmids possessed transmissible ability, and over half of the CRKP strains contained transmissible MDR plasmids. The virulence plasmids in hv-CRKP strains and hv-MDR-CRKP strains were mainly mobilizable and coexisted with conjugative MDR plasmids (Additional file 5: Fig. S13c; Additional file 3: Table S3). This indicated that most of the mobilizable plasmids might have transfer potential with the help of coexisting conjugative plasmids.

## Discussion

*K. pneumoniae* is a reservoir of plasmids. It serves as vehicle to deliver plasmids between the environment and other bacteria [51]. Previous reports found that plasmid-carrying virulence genes, including the aerobactin synthesis locus *iuc*, ropy mucoid regulator gene *rmpA2*, and cluster *rmpADC*, could transfer and confer hypervirulence to *K. pneumoniae* [16, 32–34]*.* Plasmids are also significant vehicles conferring MDR or CR phenotypes to *K. pneumoniae* [15, 17]*.* However, the role of mobilizable plasmids in facilitating the transfer of AMR and virulence genes in *K. pneumoniae* has been relatively overlooked. In this study, we observed that the naturally mobilizable MDR plasmid pKPHS3 and virulence plasmid pRJF293 without the relaxase gene could be cotransferred with the CR conjugative plasmid pKPHS2 across various *Klebsiella* and *E. coli* strains. We also found that mobilizable plasmids without the proto-spacer, the target of the CRISPR–Cas system [52], can be free of CRISPR–Cas interference in recipients. The in silico analysis of more than three thousand plasmids showed that mobilizable plasmids possessing fewer proto-spacers have a broader host range. In addition, mobilizable plasmids and conjugative plasmids together aid the enrichment of AMR genes and acquired virulence genes in *K. pneumoniae*.

The mobilizable plasmids impact HGT events in *K. pneumoniae* in a hidden and unobvious way. The lack of a relaxase gene or undiscovered *oriT*s restrains the detection and tracking of mobilizable plasmids. In the past, mobilizable virulence plasmids were often found in the hvKP strains K1 and K2 [16]. However, recently, these mobilizable virulence plasmids have been widespread in ST11 *K. pneumoniae* strains [36, 53, 54]. The majority of these prevailing transmitted virulence plasmids usually coexist or fuse with IncF conjugative plasmids [36, 55]. Apart from 89.0% (113/127) of virulence plasmids being mobilizable, our genomic analysis showed that 22.3% (248/1111) of MDR plasmids and 15.5% (93/600) of CR plasmids in *K. pneumoniae* also belong to mobilizable plasmids. The mobilizable plasmids were believed to be hardly transmissible because of their extremely low transfer frequency and limited experiments on the transfer of mobilizable plasmids [4, 5, 9]. Recent work found that more than half of plasmids in *E. coli* and *S. aureus* are mobilizable plasmids [7, 46], of which most are relaxase-absent and have the highest densities of AMR genes. In this study, conjugation assays observed the transfer of natural mobilizable virulence or MDR plasmids that have *oriT*s but lack the relaxase gene with the helper conjugative CR plasmid. By using oriTfinder [30], 528 out of 811 mobilizable plasmids of the completely sequenced *K. pneumoniae* strains were found to carry an *oriT* but not a relaxase gene. Our former work also presented that the mobilizable virulence plasmids without the relaxase gene could be transferred in *K. pneumoniae* via interaction with an IncFIB conjugative plasmid with a MOB_H_ relaxase [14] or IncFIA/IncN conjugative plasmids with a MOB_F_ relaxase [13]. In this study, we experimentally validated that the naturally mobilizable MDR plasmid pKPHS3 and virulence plasmid pRJF293 lacking the relaxase gene could be transferred with the help of the conjugative CR plasmid pKPHS2. These results indicate that mobilizable plasmids without relaxase can transfer with the aid of their *oriT* and the helper conjugative plasmid (Additional file 5: Fig. S14). In addition, we conducted growth curves and competition assays between the recipients *K. pneumoniae* RJF293 and *E. coli* C600 and their transconjugants to explore the effects of natural selection on conjugation results (Additional file 5: Fig. S15). These experiments showed that the plasmids had a slight impact on the growth of transconjugants but did not affect the results of detection on plasmid mobility in this study.

Self-transmissible conjugative plasmids are believed to have great power to transmit and a much higher transfer frequency than mobilizable plasmids [9, 14]. However, CRISPR–Cas systems usually target conserved genes coding for the conjugative apparatus and restrain the dissemination of conjugative plasmids [11, 12]. Conjugative plasmids contain most of the proto-spacers and are targeted by CRISPR–Cas systems in *K. pneumoniae*. In ST11 strains, 53.2% (383/720) of plasmids were targeted by the CRISPR–Cas systems, including 90.1% (164/182) of CR plasmids and 81.1% (228/281) of MDR plasmids. This might be the reason that CR plasmids were usually conjugative but were barely found in the strains carrying CRISPR–Cas systems, such as hvKPs of ST23 [12]. Interestingly, 27.6% of proto-spacers were located in conjugation-related genes on plasmids (Additional file 4: Table S4). Although mobilizable plasmids cannot self-transfer, they exhibited greater potential to be transmitted into strains with CRISPR–Cas systems. This might compensate for the fact that conjugative plasmids are often defended against HGT by the CRISPR–Cas system. In the background of the extensive use of antibiotics [56, 57], the transfer of AMR-mobilizable plasmids was facilitated under the pressure of antibiotics. The virulence genes on mobilizable plasmids could also confer a survival advantage to the *K. pneumoniae* clinical isolates, and in return, it would indirectly elevate the dissemination ability of these mobilizable plasmids. In addition, our results showed that the conjugative CR plasmid pKPHS2, which was targeted and degraded by the CRISPR–Cas system, still aided the transfer of the MDR plasmid pKPHS3 and virulence plasmid pRJF293 to *K. pneumoniae* KpBSI083A. The restraint on conjugative plasmids does not affect their aids in the transmission of mobilizable plasmids. This could interpret the phenomenon from one aspect some mobilizable plasmids are widespread without the accompaniment of conjugative plasmids.

## Conclusions

Our work proposed the important impact of mobilizable plasmids on the HGT of AMR genes and acquired virulence genes in *K. pneumoniae.* The mobilizable plasmid might have enormous potential to disseminate and could escape from the restraint of the CRISPR–Cas system. The cotransfer mode of mobilizable plasmids with conjugative plasmids could contribute to the cotransfer of AMR plasmids and virulence plasmids. To curtail the further cotransmission of AMR genes and virulence genes, effective surveillance and flexible strategies are needed to target the circulation of both mobilizable plasmids and conjugative plasmids.

### Supplementary Information

**Additional file 1: Table S1.** Strains and plasmids used in this study.

**Additional file 2: Table S2.** Primers and other oligonucleotides used in this study.

**Additional file 3: Table S3.** Genomic information of 1,194 completely sequenced *K. pneumoniae* strains with 3,923 plasmids from GenBank.

**Additional file 4: Table S4.** CRISPR-Cas systems, spacers, and proto-spacers detected in the complete genome sequences of *K. pneumoniae* from GenBank.

**Additional file 5:** **Supplementary Figures 1-15.**

**Additional file 6: Table S5.** Conjugation frequency of plasmids in this study.

**Additional file 7: Table S6.** Stability of plasmids in 13 days of serial passage culture.

## Data Availability

The complete genome sequences of transconjugants *E. coli* C600-p2-3 and *E. coli* C600-p2-V were deposited in the NCBI BioProject repository under the accession number PRJNA903763 (https://www.ncbi.nlm.nih.gov/bioproject/PRJNA903763/) [23]. The accession numbers of all the other genome sequences analyzed during the current study are included in this manuscript and available in the NCBI Nucleotide database [58].

## Abbreviations

HGT: Horizontal gene transfer
MDR: Multidrug resistance
CR: Carbapenem resistance
AMR: Antimicrobial resistance
cKP: Classical *K. pneumoniae*
hvKP: Hypervirulent *K. pneumoniae*
CRKP: Carbapenem-resistant *K. pneumoniae*
hv-CRKP: Hypervirulent carbapenem-resistant *K. pneumoniae*
hv-MDR-CRKP: Hypervirulent multidrug-resistant carbapenem-resistant *K. pneumoniae*
CRISPR–Cas: Clustered regularly interspersed short palindromic repeats-CRISPR- associated protein
ST: Sequence type
WGS: Whole-genome sequencing
IS: Insertion sequence element
*ori*T: Origin region of transfer
T4SS: Type IV secretion system
T4CP: Type IV coupling protein
