# Supplementary material for: Mobilizable plasmids drive the spread of antimicrobial resistance genes and virulence genes in Klebsiella pneumoniae
Source: Genome Med. 2023 Dec 1;15:106. doi: 10.1186/s13073-023-01260-w (PMC10691111; doi:10.1186/s13073-023-01260-w)
Supplement: Supplementary file 5 — Additional file 5: Supplementary Figures 1-15. [file 13073_2023_1260_MOESM5_ESM.pdf]

Mobilizable plasmids drive the spread of antimicrobial resistance genes and virulence genes in *Klebsiella pneumoniae.*

Supplementary figures

Fig. S1. The mobilizable MDR plasmid pKPHS3 can be transferred between different *K. pneumoniae* strains with the help of CR plasmid pKPHS2.

Fig. S2. The mobilizable virulence plasmid pRJF293 can be transferred between different *Klebsiella* strains with the help of CR plasmid pKPHS2.

Fig. S3. MDR plasmid pKPHS3 and virulence plasmid pRJF293 can be transferred into *E. coli* C600 in various patterns with the help of CR plasmid pKPHS2.

Fig. S4. Genetic structure of the fusion pKPHS2-3 of the transconjugant *E. coli* C600-p2-3.

Fig. S5. Genetic structure of the fusion plasmids p2-V-1 and p2-V-2 of the transconjugant *E. coli* C600-p2-V.

Fig. S6. Stabilization of hybrid plasmid in transconjugant detected by S1-PFGE.

Fig. S7 The mobilizable virulence plasmid pRJF293 and the mobilizable MDR plasmid pKPHS3 can be transferred into KpBSI083A and its derivate KpBSI083*Δcas3*H with the help of conjugative CR plasmid pKPHS2 (p2).

Fig. S8. Proposed mechanism of KpBSI083A-p3 and KpBSI083A-pV generation.

Fig. S9. Distribution of CRISPR–Cas systems and plasmids in 1,194 completely sequenced *K. pneumoniae* strains taken from GenBank.

Fig. S10. The number of plasmids targeted and nontargeted by endogenous CRISPR–Cas systems.

Fig. S11. Distribution of conjugative, mobilizable, and nonmobilizable plasmids according to plasmid size in six typical STs of *K. pneumoniae* strains.

Fig. S12. Distribution and mobility of clinically alarming plasmids in *K. pneumoniae*.

Fig. S13. Coexistence of plasmids in 1,194 *Klebsiella pneumoniae* strains.

Fig. S14. Proposed transfer interaction of the mobilizable plasmid and conjugative plasmid.

Fig. S15. Growth curves and competition assays of recipients *K. pneumoniae* RJF293, *E. coli* C600 and their transconjugants.

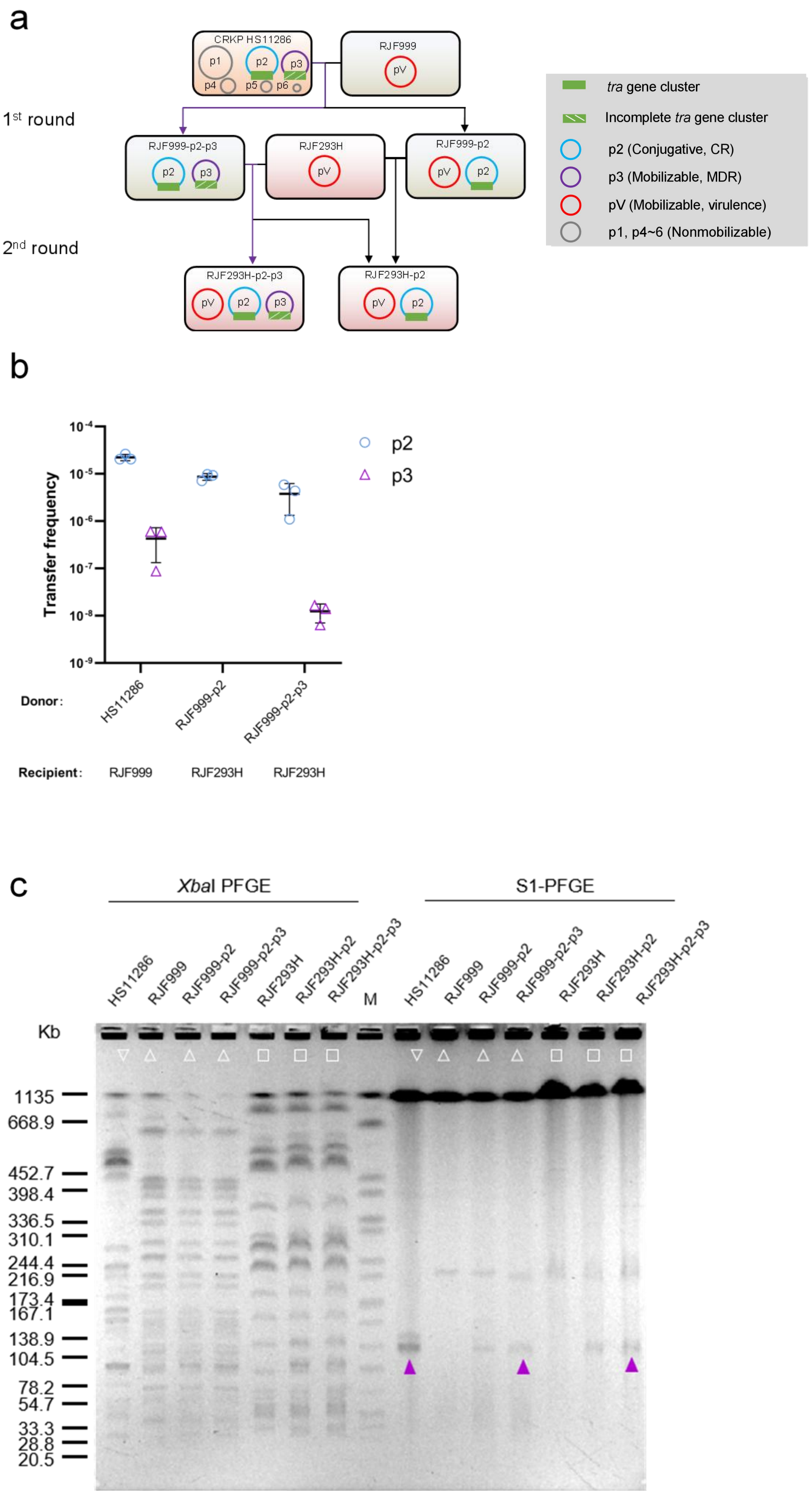
a
CRKP HS11286
p1
p2
p3
p4
p5
p6
RJF999
pV
1st round
RJF999-p2-p3
RJF293H
RJF999-p2
2nd round
RJF293H-p2-p3
RJF293H-p2
tra gene cluster
Incomplete tra gene cluster
p2 (Conjugative, CR)
p3 (Mobilizable, MDR)
pV (Mobilizable, virulence)
p1, p4~6 (Nonmobilizable)
b
Transfer frequency
10-4
10-5
10-6
10-7
10-8
10-9
p2
p3
Donor:
HS11286
RJF999-p2
RJF999-p2-p3
Recipient:
RJF999
RJF293H
RJF293H
c
XbaI PFGE
S1-PFGE
HS11286
RJF999
RJF999-p2
RJF999-p2-p3
RJF293H
RJF293H-p2
RJF293H-p2-p3
M
Kb
1135
668.9
452.7
398.4
336.5
310.1
244.4
216.9
173.4
167.1
138.9
104.5
78.2
54.7
33.3
28.8
20.5

Fig. S1. The mobilizable MDR plasmid pKPHS3 (p3) can be transferred between different *K. pneumoniae* strains with the help of CR plasmid pKPHS2 (p2). (**a**) Schematic diagram of the conjugation assays. The green square denotes the *tra* gene cluster on pKPHS2. The green square with white slashes denotes the incomplete *tra* gene cluster on pKPHS3. (**b**) The conjugation frequencies of pKPHS2 and pKPHS3. The donor strains are HS11286, RJF999-p2 and RJF999-p2-p3. The recipient strains are RJF999 and RJF293H. Detailed data are available in Additional file 6: Table S5. (**c**) *Xba*I PFGE and S1-PFGE of transconjugants and their parental strains. M represents the molecular weight marker, *Salmonella* serotype *Braenderup* H9812 strain. Strains with the same symbol on the PFGE image represent the progeny derived from the same parental strain. The purple triangle denotes the location of the band of pKPHS3 and the strain harboring pKPHS3 is also validated by PCR.

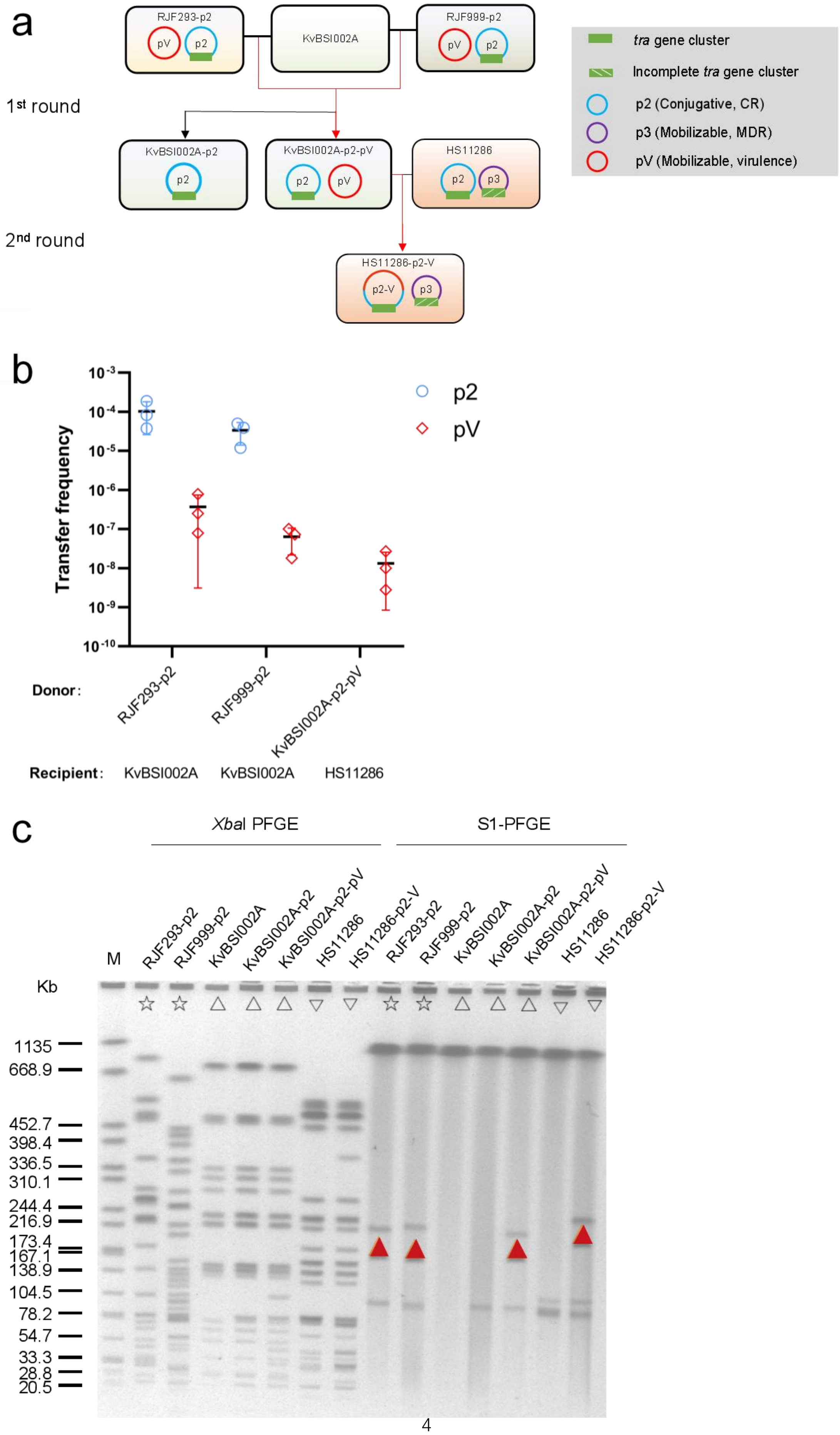

a
1st round
2nd round
RJF293-p2
KvBSI002A
RJF999-p2
KvBSI002A-p2
KvBSI002A-p2-pV
HS11286
HS11286-p2-V
tra gene cluster
Incomplete tra gene cluster
p2 (Conjugative, CR)
p3 (Mobilizable, MDR)
pV (Mobilizable, virulence)
b
Transfer frequency
p2
pV
Donor:
RJF293-p2
RJF999-p2
KvBSI002A-p2-pV
Recipient:
KvBSI002A
KvBSI002A
HS11286
c
XbaI PFGE
S1-PFGE
M
Kb
1135
668.9
452.7
398.4
336.5
310.1
244.4
216.9
173.4
167.1
138.9
104.5
78.2
54.7
33.3
28.8
20.5

Fig. S2. The mobilizable virulence plasmid pRJF293 (pV) can be transferred between different *Klebsiella* strains with the help of CR plasmid pKPHS2 (p2). (**a**) Schematic diagram of the conjugation assays. The green square denotes the *tra* gene cluster on pKPHS2. The green square with white slashes denotes the incomplete *tra* gene cluster on pKPHS3 (p3). (**b**) The conjugation frequencies of pKPHS2 and pRJF293. The donor strains are RJF999-p2, RJF293-p2 and KvBSI002A-p2-pV. The recipient strains are KvBSI002A and HS11286. Detailed data are available in Additional file 6: Table S5. (**c**) *Xba*I PFGE and S1-PFGE of transconjugants and their parental strains. M represents the molecular weight marker, *Salmonella* serotype *Braenderup* H9812 strain. Strains with the same symbol on the PFGE image represent the progeny derived from the same parental strain. The purple triangle denotes the location of the band of pKPHS3 and the strain harboring plasmid pV is also validated by PCR.

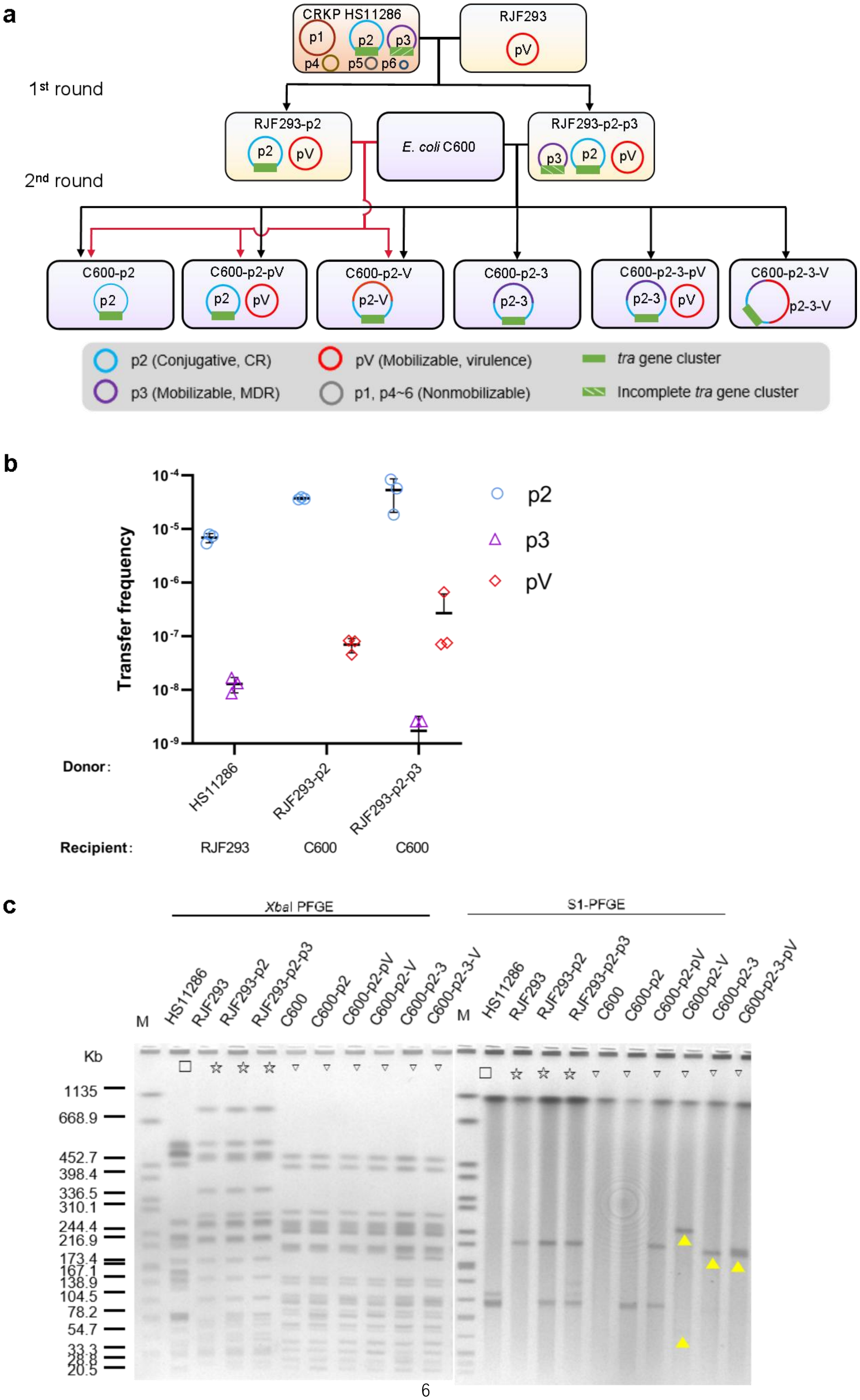
a
CRKP HS11286
p1
p2
p3
p4
p5
p6
RJF293
pV
1st round
RJF293-p2
E. coli C600
RJF293-p2-p3
2nd round
C600-p2
C600-p2-pV
C600-p2-V
C600-p2-3
C600-p2-3-pV
C600-p2-3-V
p2-V
p2-3
p2-3-V
p2 (Conjugative, CR)
pV (Mobilizable, virulence)
tra gene cluster
p3 (Mobilizable, MDR)
p1, p4~6 (Nonmobilizable)
Incomplete tra gene cluster
b
Transfer frequency
10^-4
10^-5
10^-6
10^-7
10^-8
10^-9
p2
p3
pV
Donor:
HS11286
RJF293-p2
RJF293-p2-p3
Recipient:
RJF293
C600
C600
c
XbaI PFGE
S1-PFGE
M
HS11286
RJF293
RJF293-p2
RJF293-p2-p3
C600
C600-p2
C600-p2-pV
C600-p2-V
C600-p2-3
C600-p2-3-V
C600-p2-3-pV
Kb
1135
668.9
452.7
398.4
336.5
310.1
244.4
216.9
173.4
167.1
138.9
104.5
78.2
54.7
33.3
28.8
20.5

Fig. S3. MDR plasmid pKPHS3 (p3) and virulence plasmid pRJF293 (pV) transfer into *E. coli* C600 in various patterns with the help of CR plasmid pKPHS2 (p2). (**a**) Schematic diagram of the conjugation assays. The green square denotes the *tra* gene cluster on pKPHS2. The green square with white slashes denotes the incomplete *tra* gene cluster on pKPHS3. The orange line indicates the transconjugants generated from *K. pneumoniae* RJF293-p2 conjugated with *E. coli* C600. (**b**) The conjugation frequencies of pKPHS2, pKPHS3 and pRJF293. The donor strains are HS11286, RJF293-p2 and RJF293-p2-p3. The recipient strains are C600 and RJF293. Detailed data are available in Additional file 6: Table S5. (**c**) *Xba*I PFGE and S1-PFGE of transconjugants and their parental strains. M represents the molecular weight marker, *Salmonella* serotype *Braenderup* H9812 strain. Strains with the same symbol on the PFGE image represent the progeny derived from the same parental strain. The yellow triangle denotes the hybrid plasmid.

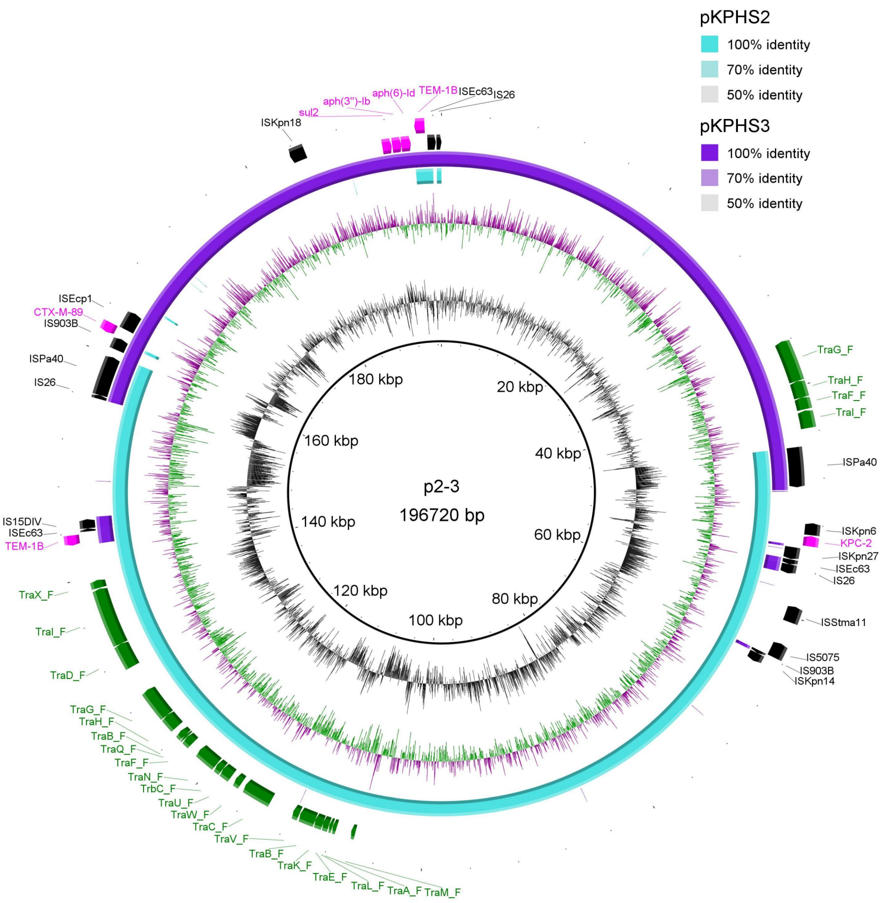

Fig. S4. Genetic structure of the fusion p2-3 of the transconjugant *E. coli* C600-p2-3. The *tra* genes are indicated in green, IS elements are shown in black and resistance genes are shown in fuchsia. The hybrid p2-3 has a length of 196,720 bp and a GC content of 52%.

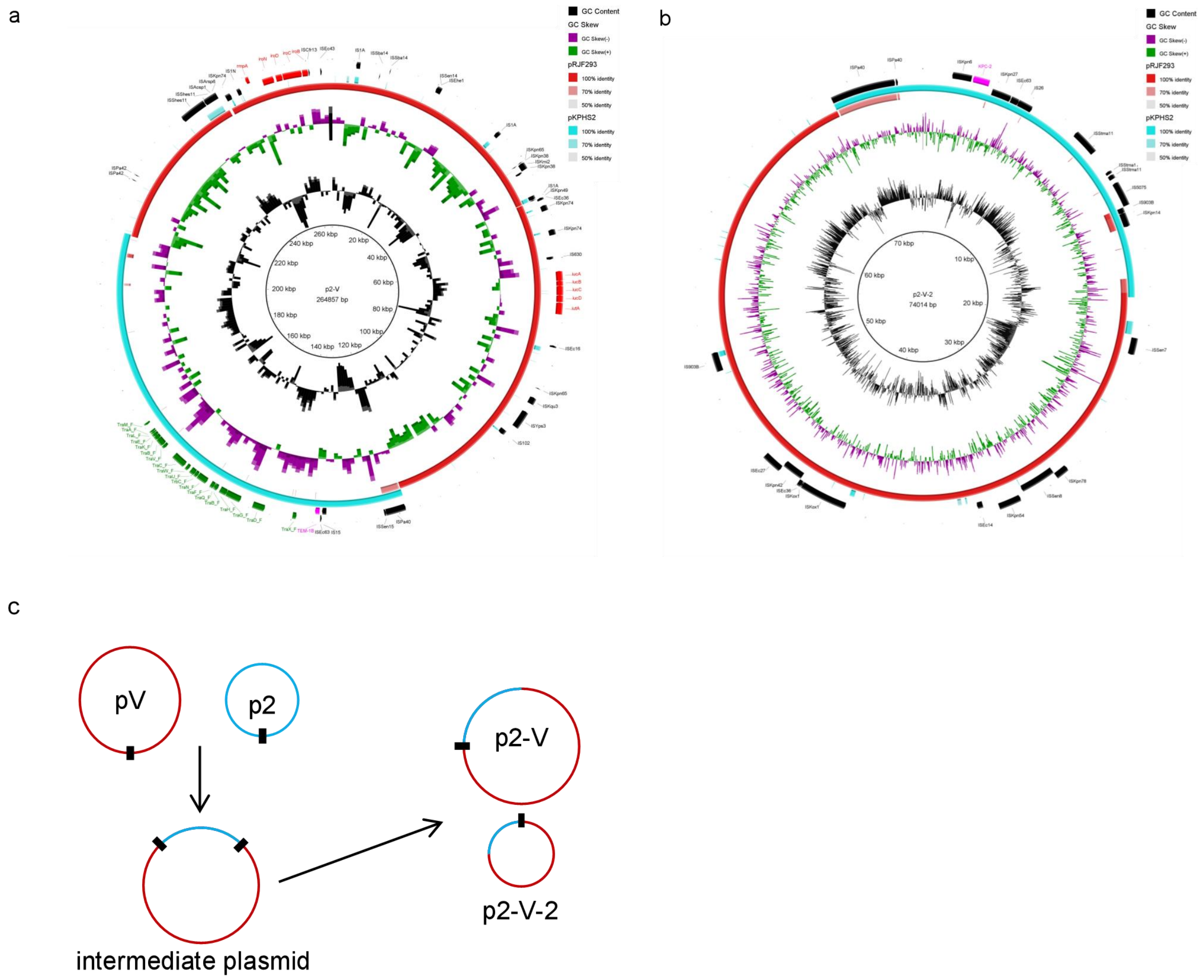

Fig. S5. Genetic structure of the fusion plasmids p2-V-1 and p2-V-2 of the transconjugant *E. coli* C600-p2-V. (**a**) The p2-V has a length of 264,857 bp and a CG content of 51% and contains *tra* genes and virulence genes. (**b**) The p2-V-2 has a length of 74,014 bp and a CG content of 54% and contains the carbapenemase gene bla_{KPC}. The *tra* genes are indicated in green. Virulence genes are indicated in red. IS elements are shown in black. Resistance genes are shown in fuchsia. (**c**) The speculative process of formation of p2-V and p2-V-2. The red circle presents the backbone of plasmid pV and the blue circle presents the backbone of pKPHS2 (p2). The black band is the region of IS*pa40*.

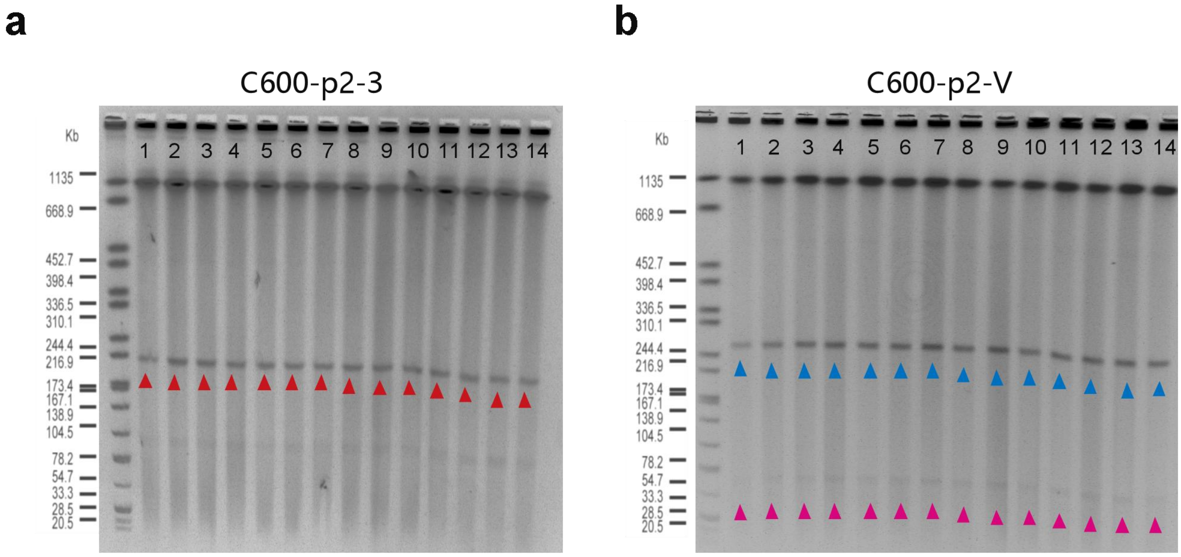

Fig. S6. Stabilization of hybrid plasmid in transconjugant detected by S1-PFGE. (**a**) Stabilization of hybrid p2-3 in C600-p2-3. (**b**) Stabilization of hybrid plasmids in C600-p2-V. M represents the molecular weight marker, *Salmonella* serotype *Braenderup* H9812 strain. Red triangles denote the fusion p2-3. Blue triangles denote the fusion pKPHS2-V and fuchsia triangles denote the fusion p2-V-2.

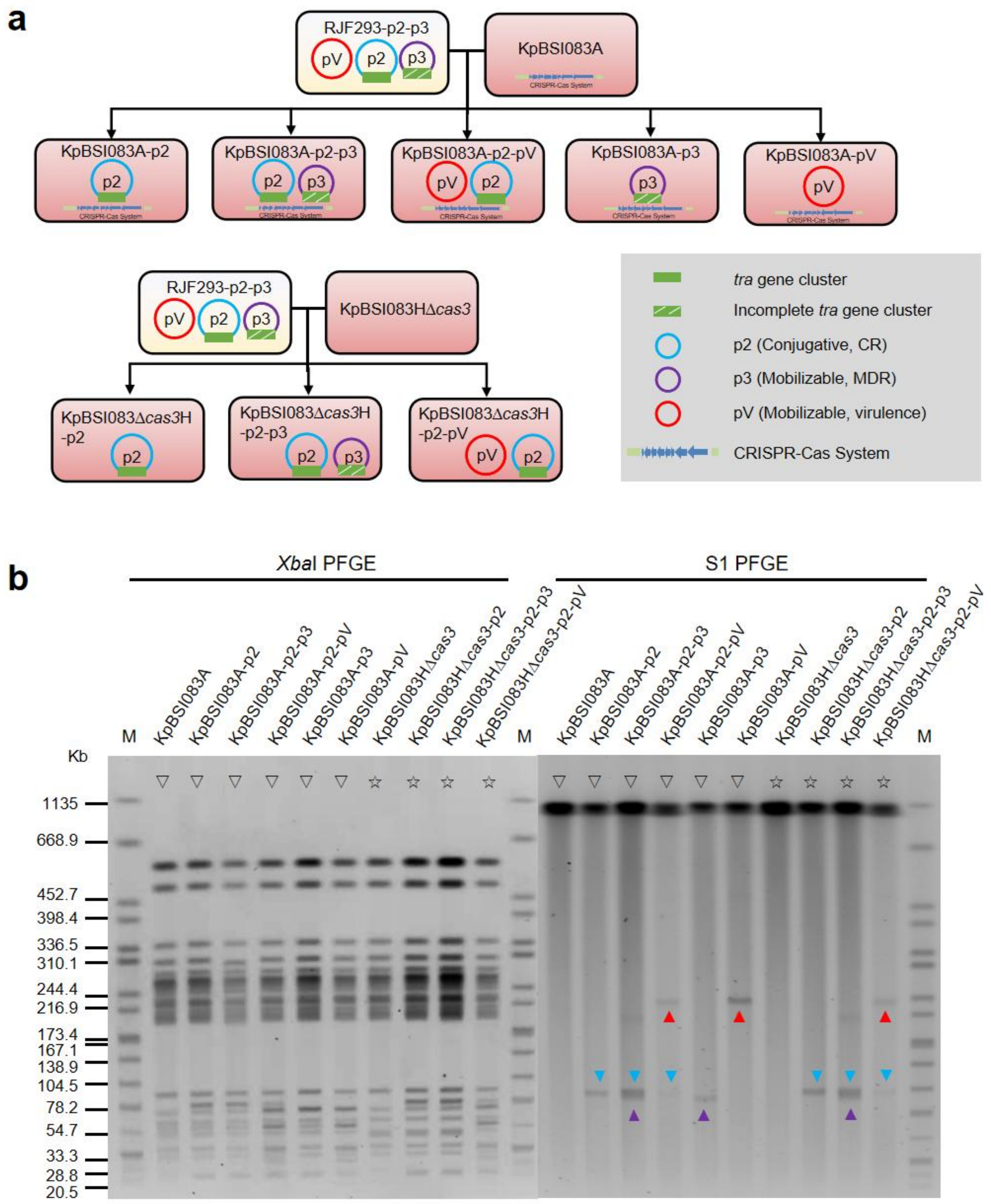

Fig. S7 The mobilizable virulence plasmid pRJF293 (pV) and the mobilizable MDR plasmid pKPHS3 (p3) can be transferred into KpBSI083A and its derivate KpBSI083*Δcas3*H with the help of conjugative CR plasmid pKPHS2 (p2). (a) Schematic diagram of the conjugation assays. The green square denotes the *tra* gene cluster on pKPHS2. The green square with white slashes denotes the incomplete *tra* gene cluster on pKPHS3. (a) *Xba*I PFGE and S1-PFGE of transconjugants and their parental strains. M represents the molecular weight marker, *Salmonella* serotype *Braenderup* H9812 strain. Strains with the same symbol on the PFGE image represent the progeny derived from the same parental strain.

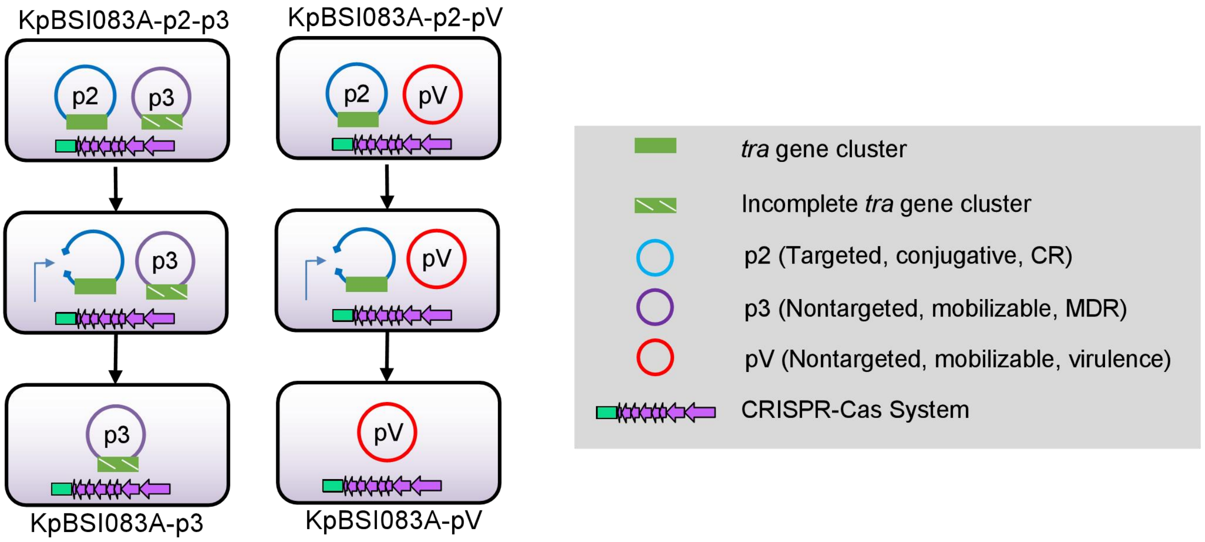

Fig. S8. Proposed mechanism of KpBSI083A-p3 and KpBSI083A-pV generation. During the bacterial growth phase, transconjugants KpBSI083A-p2-p3 and KpBSI083A-p2-pV experienced pKPHS2 (p2) loss due to the interference of the CRISPR–Cas system. Subsequently, these strains evolved into KpBSI083A-p3 and KpBSI083A-pV, respectively.

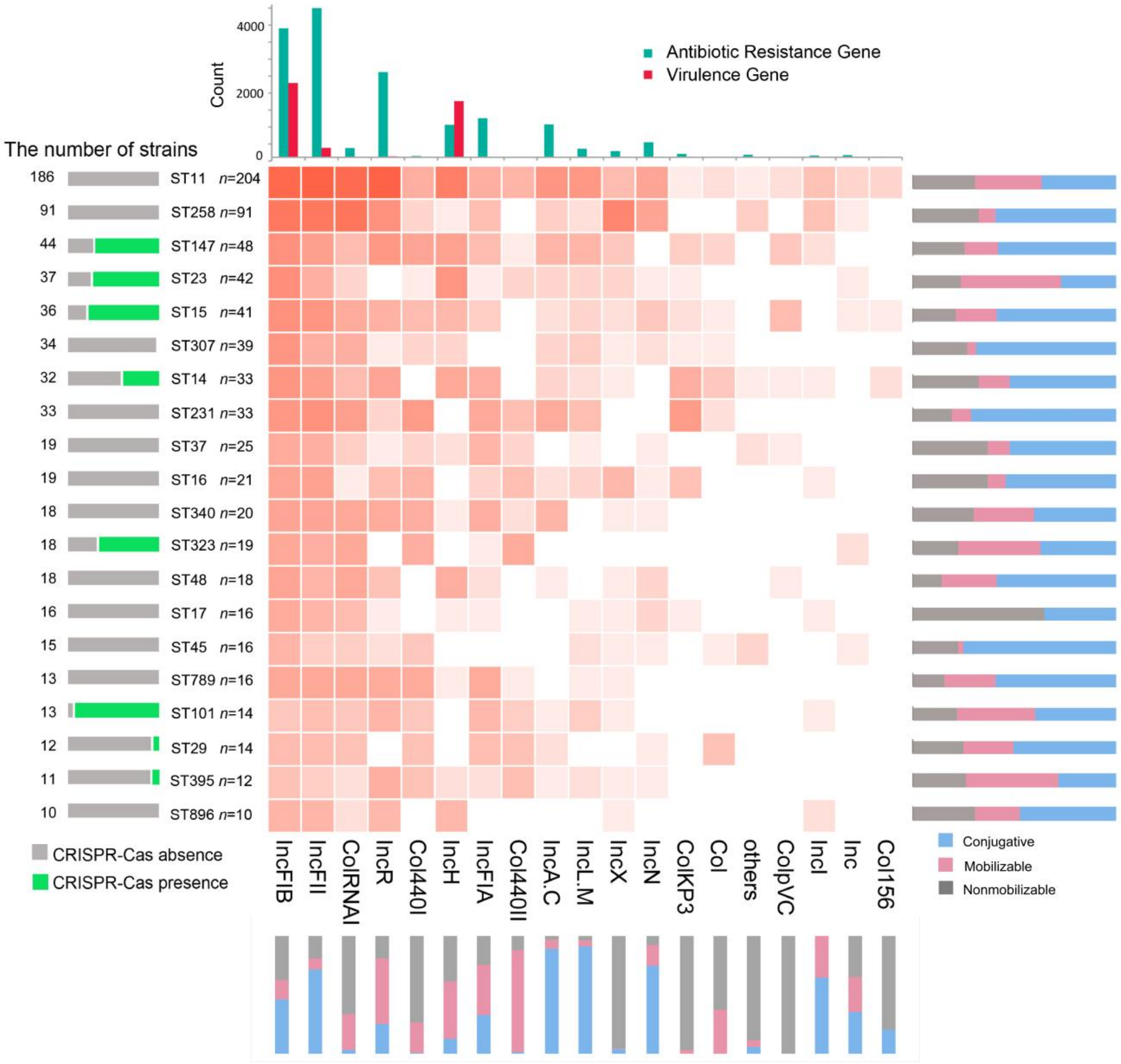

Fig. S9. Distribution of CRISPR–Cas systems and plasmids in 1,194 completely sequenced *K. pneumoniae* strains taken from GenBank. The left bar chart shows the proportion of *K. pneumoniae* strains in the presence of the CRISPR–Cas system. The green bar indicates the *K. pneumoniae* strains containing CRISPR–Cas systems. The middle heatmap shows the distribution of plasmids with different replicons in the *K. pneumoniae* strains of the top 20 STs associated with the highest number of plasmids. The right bar chart presents the proportion of plasmids with different transferable abilities. The top bar chart shows the number of resistance genes and acquired virulence genes per plasmid with different replicons. The bottom bar chart indicates the scale of plasmid mobility in plasmids with different replicons.

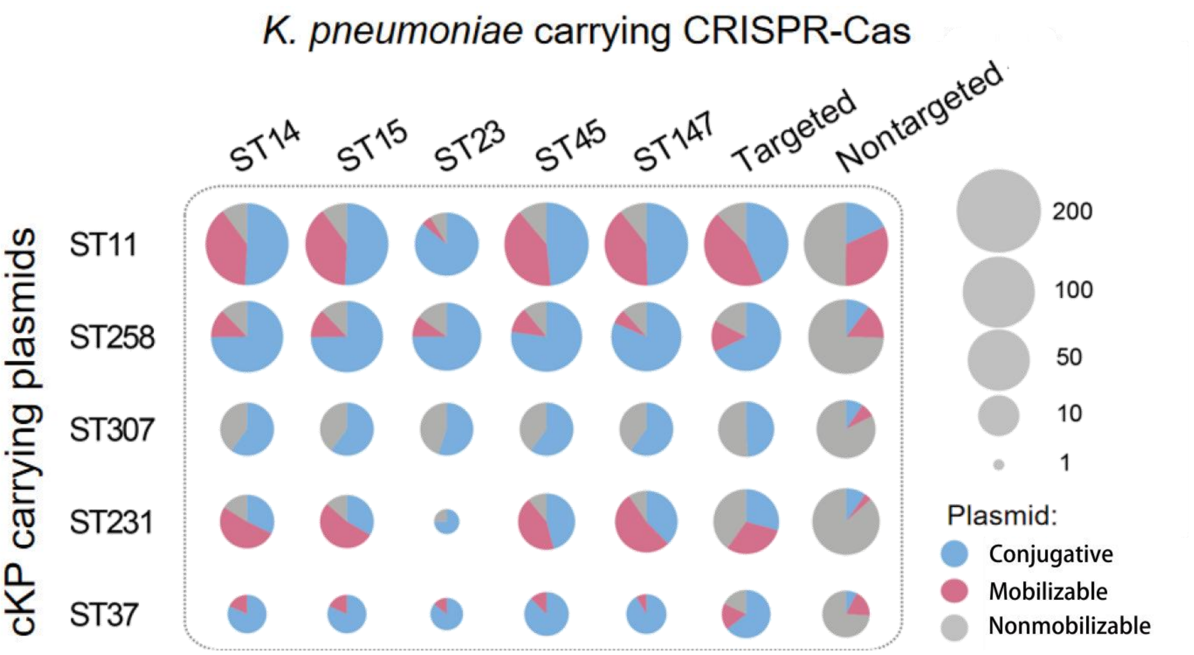

Fig. S10. The number of plasmids targeted and nontargeted by endogenous CRISPR – Cas systems. From left to right, the first five columns represent the plasmids from the cKP strains (ST11, ST258, ST307, ST231, and ST37) without CRISPR – Cas system are targeted by CRISPR – Cas systems of *K. pneumoniae* from strains (ST14, ST15, ST23, ST45, and ST147). The sixth column represents the plasmids in those strains targeted by all CRISPR–Cas systems in *K. pneumoniae*. The last column represents the plasmids without targets of the CRISPR – Cas system in *K. pneumoniae*. The number of plasmids corresponds to the size of the pie charts.

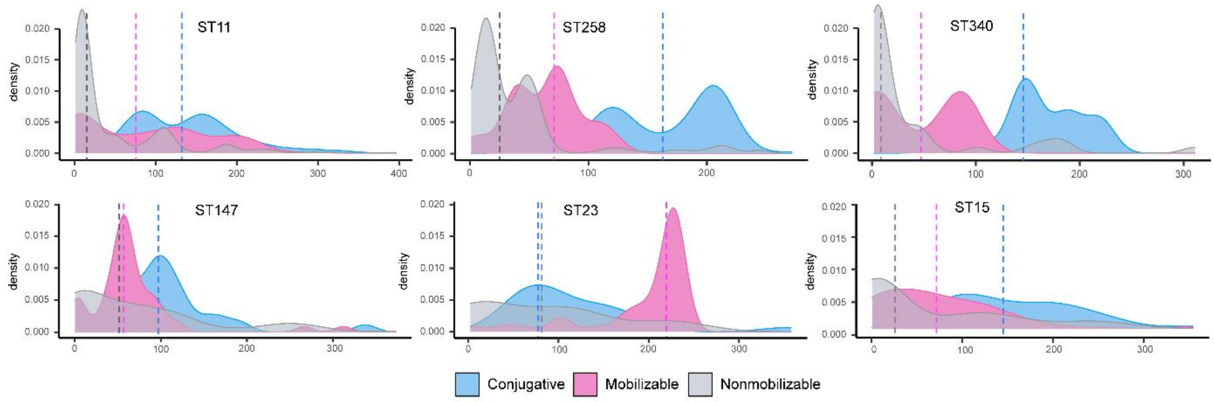

Fig. S11. Distribution of conjugative, mobilizable, and nonmobilizable plasmids according to plasmid size in six typical Sequence Types of *K. pneumoniae* strains*.* Six typical STs ranging from ST11 to ST15 were selected to investigate the density and distribution of plasmids. The medians of plasmid size of conjugative plasmid, mobilizable plasmid, and nonmobilizable plasmid were presented via blue, pink, and gray dotted lines, respectively.

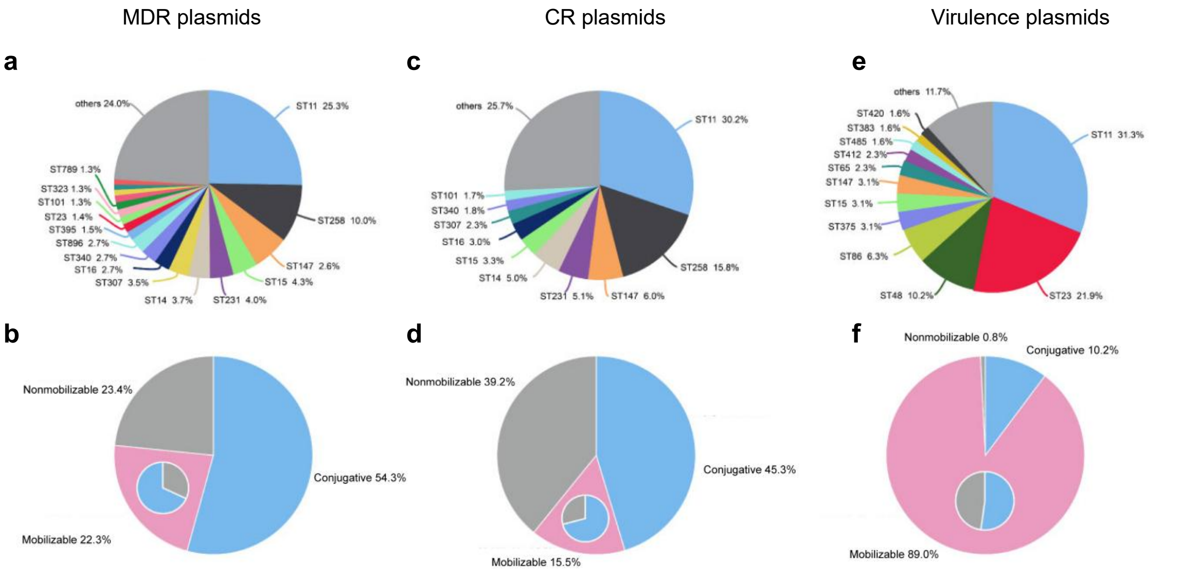

Fig. S12. Distribution of clinically alarming plasmids in *K. pneumoniae*. (**a**) MDR plasmids carried by different MLST host strains. (**b**) Mobilities of MDR plasmids. (c) CR plasmids carried by different MLST host strains. (**d**) Mobilities of CR plasmids. (**e**) Virulence plasmids carried by different MLST host strains. (**f**) Mobilities of virulence plasmids. The proportion of mobilizable plasmids that co-occurred with (blue) or without (gray) conjugative plasmids was indicated *via* small pie charts.

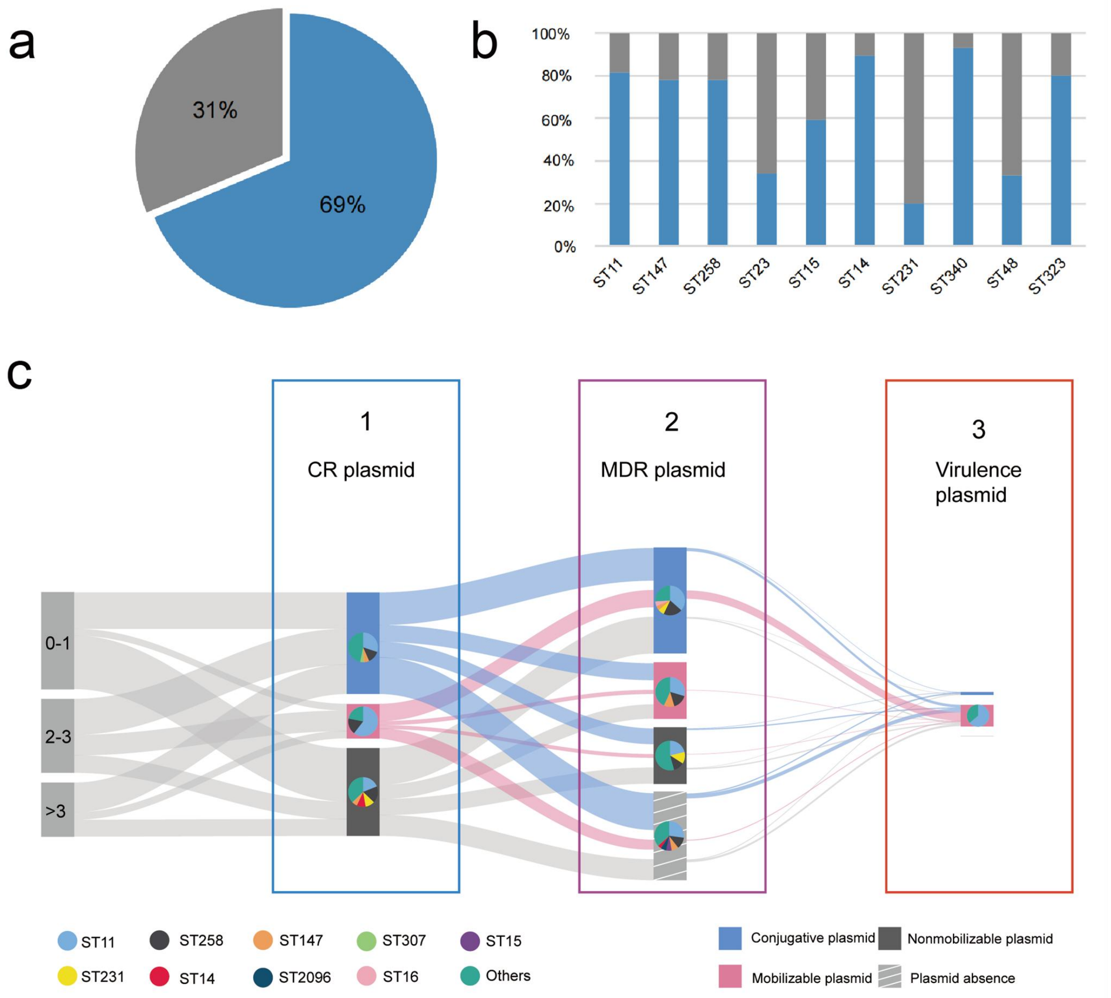

Fig. S13. Coexistence of plasmids in 1,194 *Klebsiella pneumoniae* strains. (**a**) The pie chart presents the proportion of mobilizable plasmids that coexist with conjugative plasmids in *K. pneumoniae* strains. (**b**) The histogram indicates the proportion of mobilizable plasmids that coexist with conjugative plasmids in the *K. pneumoniae* strains of the top 10 STs associated with the number of plasmids. The blue indicated the mobilizable plasmids which coexist with conjugative plasmids. The gray presented the mobilizable plasmids that exist alone. (**c**) The coexistence of CR plasmids, MDR plasmids, and virulence plasmids in 537 CRKP strains. The first column indicates 600 CR plasmids with different mobilities in 537 CRKP strains and the left connected gray flow indicates the additional AMR genes on CR plasmids. Conjugative plasmids are indicated by blue, mobilizable plasmids are indicated by pink, and nonmobilizable plasmids are marked by gray. The second column shows that CRKP strains harbour MDR plasmids with different mobilities or contain no MDR plasmids. In the second column, CRKP strains without MDR plasmids are indicated by connecting the flow to the gray rectangles with white slashes. The third column indicates virulence plasmids in CRKP strains. The distribution of CRKP strains with different plasmids in different STs of *K. pneumoniae* is presented by a pie chart on each column and the information on ST is indicated at the bottom right. The breadth of flow between different columns depicts the number of coexistence of plasmids in CRKP strains.

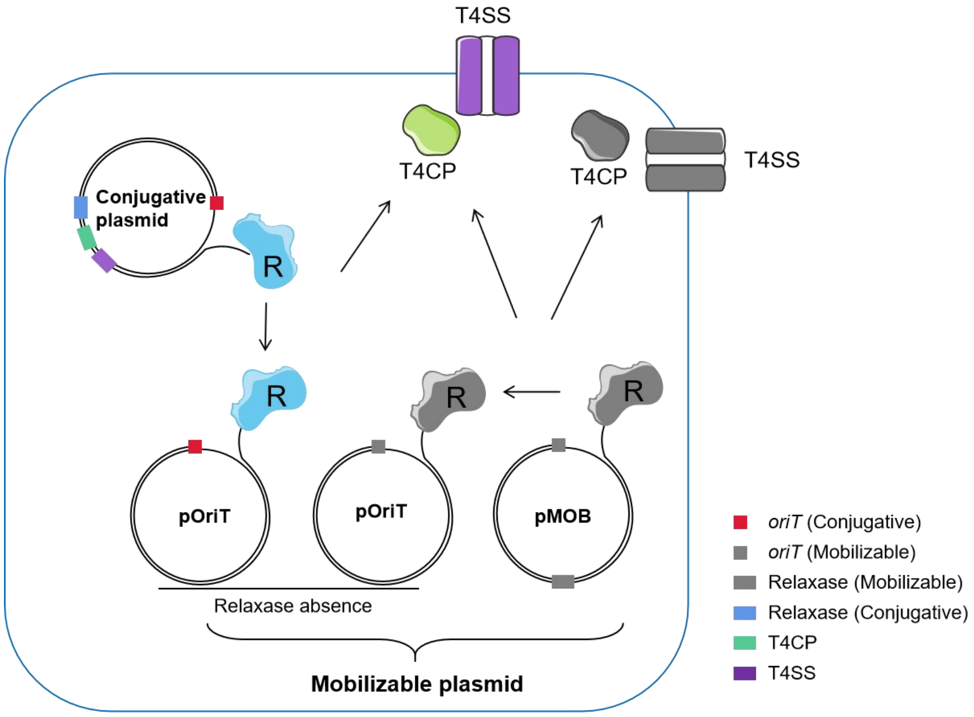

Fig. S14. Proposed transfer interaction of the mobilizable plasmid and conjugative plasmid. The conjugative plasmids encode four modules for plasmid conjugation, including the relaxase which could recognize and nick *oriT* on plasmid. The mobilizable plasmids carrying both an *oriT* and a relaxase gene (pMOB) can transfer with the help of conjugative elements encoded by conjugative plasmids. The mobilizable plasmids carrying an *oriT* but lacking a relaxase gene (pOriT) could transfer under the help of relaxase encoded by other plasmids and the aid of conjugative elements supported by the conjugative plasmid.

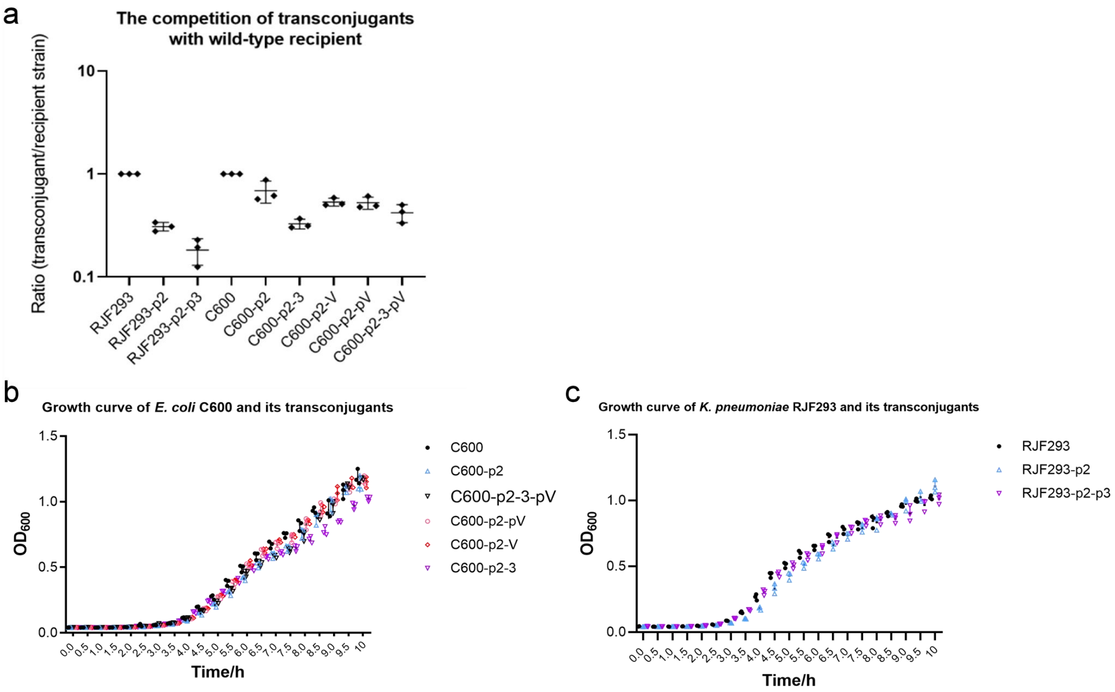

Fig. S15. **The growth curves and competition assays of recipients *K. pneumoniae* RJF293, *E. coli* C600 and their transconjugants**. (**a**) Competition assays between recipients *K. pneumoniae* RJF293 or *E. coli* C600 with their transconjugants in this study. (**b**) The growth curves of recipient strain *E. coli* C600 and its transconjugants, including C600-p2, C600-p2-3, C600-p2-V, C600-p2-3-pV, and C600-p2-pV. (**c**) The growth curves of recipient strain *K. pneumonia* RJF293, and its transconjugants including RJF293-p2 and RJF293-p2-p3.
